# Antimicrobial Usage Among Acutely Ill Hospitalized Children Aged 2‒23 Months in Sub-Saharan Africa and South Asia

**DOI:** 10.1093/ofid/ofaf487

**Published:** 2025-08-18

**Authors:** Caroline Tigoi, Celine Bourdon, Moses Ngari, Robert Musyimi, Molly Timbwa, Shalton Mwaringa, Narshion Ngao, Christopher Maronga, Moses Mburu, Agnes Ndirangu, Fehmina Arif, Zaubina Kazi, Muzammil Shabana Ejaz, Ali Faisal Saleem, Benson O Singa, Ezekiel Mupere, Abu Sadat Mohammad Sayeem Bin Shahid, Al Fazal Khan, Mohammod Jobayer Chisti, Tahmeed Ahmed, Christina Lancioni, Abdoulaye Diallo, Wieger Voskuijl, Robert H Bandsma, Kirkby D Tickell, Priya Sukhtanar, Judd L Walson, Nicole Stoesser, James A Berkley

**Affiliations:** Nuffield Department of Medicine, University of Oxford, Oxford, UK; KEMRI/Wellcome Trust Research Programme, Kilifi, Kenya; Centre for Global Child Health, Hospital for Sick Children, Toronto, Ontario, Canada; KEMRI/Wellcome Trust Research Programme, Kilifi, Kenya; KEMRI/Wellcome Trust Research Programme, Kilifi, Kenya; KEMRI/Wellcome Trust Research Programme, Kilifi, Kenya; KEMRI/Wellcome Trust Research Programme, Kilifi, Kenya; KEMRI/Wellcome Trust Research Programme, Kilifi, Kenya; KEMRI/Wellcome Trust Research Programme, Kilifi, Kenya; KEMRI/Wellcome Trust Research Programme, Kilifi, Kenya; KEMRI/Wellcome Trust Research Programme, Kilifi, Kenya; Department of Pediatrics and Child Health, Aga Khan University, Karachi, Pakistan; Department of Pediatrics and Child Health, Aga Khan University, Karachi, Pakistan; Department of Pediatrics and Child Health, Aga Khan University, Karachi, Pakistan; Department of Pediatrics and Child Health, Aga Khan University, Karachi, Pakistan; Centre for Clinical Research, Kenya Medical Research Institute, Nairobi, Kenya; Department of Paediatrics and Child Health, Makerere University College of Health Sciences, Kampala, Uganda; International Centre for Diarrhoeal Disease Research, Bangladesh (icddr,b), Dhaka, Bangladesh; International Centre for Diarrhoeal Disease Research, Bangladesh (icddr,b), Dhaka, Bangladesh; International Centre for Diarrhoeal Disease Research, Bangladesh (icddr,b), Dhaka, Bangladesh; International Centre for Diarrhoeal Disease Research, Bangladesh (icddr,b), Dhaka, Bangladesh; Department of Pediatrics, Oregon Health and Science University, Portland, Oregon, USA; Department of Public Health, University Joseph Ki-Zerbo, Ouagadougou, Burkina Faso; Department of Biomedical Sciences, Kamuzu University of Health Sciences, Blantyre, Malawi; Amsterdam Centre for Global Child Health & Emma Children's Hospital, Amsterdam University Medical Centres, Amsterdam, The Netherlands; Centre for Global Child Health, Hospital for Sick Children, Toronto, Ontario, Canada; Department of Biomedical Sciences, Kamuzu University of Health Sciences, Blantyre, Malawi; Translational Medicine, The Hospital for Sick Children (SickKids), Toronto, Ontario, Canada; Department of Global Health, University of Washington, Seattle, Washington, USA; KEMRI/Wellcome Trust Research Programme, Kilifi, Kenya; Department of International Health, Bloomberg School of Public Health, Johns Hopkins University, Baltimore, Maryland, USA; Nuffield Department of Medicine, University of Oxford, Oxford, UK; NIHR Oxford Biomedical Research Centre, University of Oxford, Oxford, UK; Nuffield Department of Medicine, University of Oxford, Oxford, UK; KEMRI/Wellcome Trust Research Programme, Kilifi, Kenya

**Keywords:** “broad spectrum”, access, Africa, antimicrobials, Asia

## Abstract

**Background:**

Understanding patterns of antimicrobial use is critical to supporting antibiotic stewardship and limiting antimicrobial resistance (AMR). We aimed to describe antimicrobial prescribing in acutely ill hospitalized children aged 2–23 months across a range of rural and urban hospital settings in Sub-Saharan Africa and South Asia.

**Methods:**

The Childhood Acute Illness & Nutrition (CHAIN) cohort collected data daily throughout hospitalization from children with acute illness aged 2–23 months admitted to 9 hospitals from November 2016 to January 2019. We determined proportions of children receiving antimicrobials, inpatient-days receiving antimicrobials, antimicrobial classes, World Health Organization (WHO) Access, Watch, and Reserve (AWaRe) classifications, and examined factors associated with Watch antimicrobial use.

**Results:**

Of 3101 admissions, 1422 (46%) received antimicrobials before hospitalization. A total of 2816 (91%) children received antimicrobials during 19 398/21 807 (93%) inpatient child-days. Two thousand four hundred seventy-seven (76%), 1092 (35%), and 12 (0.3%) children received Access, Watch, and Reserve antimicrobials, mostly within 48 hours of admission. Three hundred forty-one (11%) admissions received an antimicrobial without any indication. Prior admission, chronic illness, diagnoses of sepsis or meningitis, hypoglycemia, and duration of admission were associated with receiving Watch antimicrobials, while WHO danger signs, severe malnutrition, HIV, and receipt of prior antimicrobials were not, despite their known association with mortality and AMR.

**Conclusions:**

Antimicrobial use was similar across sites, with some overuse and notably limited escalation and de-escalation, likely due to guideline adherence. Guidelines need updating for the absence of relevant antimicrobial sensitivities, to include risk-based antimicrobial prescribing considering mortality risk and prior exposure to antimicrobials and the hospital environment. Hence, clinical trials of risk-differentiated care are needed.

Antimicrobials are the most prescribed medications in hospitals; however, antimicrobial resistance (AMR) and limited new antimicrobial agents pose serious threats to public health. Worldwide, AMR contributes to >1.2 million deaths annually, with the highest mortality burden in Sub-Saharan Africa [1]. Up to one-third of child deaths may be attributable to AMR [1, 2], while lack of access to antimicrobials also causes mortality [3]. The World Health Organization (WHO) Global Antimicrobial Resistance And Surveillance System (GLASS) provides a standardized approach for collection, analysis, and data sharing, supporting national AMR surveillance and interventions [4]. The WHO has classified antimicrobial agents as Access, Watch and Reserve (AWaRe) based on their intended target usage and impact on AMR [5].

Many hospitals in low- and middle-income countries (LMICs) aim to follow WHO or national guidelines, which recommend adjustment according to local susceptibilities. However, treatment decisions are mostly made without any microbiology or relevant local antibiograms. Guidelines are explicit on first-line treatment and, sometimes, second-line choices. However, they mostly leave open to clinicians the criteria and timing for escalation and de-escalation and alternatives for second- and third-line agents, and they lack guidance when children meet criteria for >1 syndrome [6, 7]. Hence there is still considerable scope for variation in practice within guidelines. This, as well as diagnostic uncertainty, can lead to excessive broad-spectrum prescribing, prolonged treatment, and inappropriate antimicrobial switching [8]. Additionally, agents to treat resistant infections may be unaffordable or unavailable [9, 10].

Most studies of pediatric inpatient antimicrobial usage utilize point prevalence surveys, determining usage and indications on a single day [5]. A recent systematic review among African children and adults suggests overuse of Watch and relative neglect of Access agents [11]. However, such studies often overrepresent private, referral, and intensive care settings, which may limit generalizability. The increasing use of Watch group antibiotics in LMICs is concerning due to their strong link to multidrug-resistant organisms (MDROs) [12]. Their use has risen sharply over the past decade [13], despite WHO recommendations that at least 60% of national antibiotic consumption should come from the Access category [14]. Reducing Watch antibiotic use and prioritizing Access agents are essential for effective antimicrobial stewardship.

We aimed to describe daily in-hospital antimicrobial use among acutely ill children aged 2–23 months at 9 hospitals in sub-Saharan Africa and South Asia [15]. We specifically examined the variation in timing and use of second- and third-line agents, for which there is limited guidance.

## METHODS

### Study Design and Population

This was a secondary analysis of the prospective Childhood Acute Illness & Nutrition (CHAIN) Network cohort, November 2016 through January 2019 [15]. CHAIN aimed to determine pathways to mortality in hospital and after discharge among acutely ill children despite provision of care according to current guidelines at 9 hospitals in Sub-Saharan Africa and South Asia: Dhaka ICDDR,B (tertiary) and Matlab ICDDR,B (secondary) in Bangladesh; Banfora Referral Hospital (secondary) in Burkina Faso; Kilifi County, Mbagathi County, and Migori County Hospitals (secondary) in Kenya; Queen Elizabeth Central Hospital (tertiary) in Malawi; Civil Hospital (tertiary) in Pakistan; and Mulago Hospital (tertiary) in Uganda. Sites were selected to represent a range of facilities, populations, and comorbidities such as malaria and HIV. Dhaka, Matlab, Kilifi County, Mbagathi County Hospitals, and Civil Hospital did blood cultures routinely. Civil Hospital and Dhaka Hospital had pediatric intensive care units. All sites had access to recommended first-line and second-line antibiotics.

CHAIN recruited acutely ill children aged 2–23 months, excluding those requiring immediate resuscitation, those who were intolerant of oral feeds before illness, and those with terminal illness, conditions requiring surgery within 6 months, likely chromosomal abnormality, poisoning, trauma, or unwillingness to remain in follow-up for 6 months. To ensure representation across a range of mortality risks, children were enrolled in 3 strata (2:1:2) by nutritional status (Supplementary Appendix 1). Patients were screened on specified days each week until the weekly quota for each stratum was met, controlling the enrollment rate to help ensure data completeness and quality [15].

Before initiation, sites were audited and supported to provide standard of care to national and/or WHO guidelines [16]. Sites were given biannual reports on clinical care including antimicrobial prescription patterns.

### Patient Consent

Written informed consent was obtained from all parents or caregivers. The Oxford University Tropical Research Ethics Committee (OXTREC 34-16) and the ethics committees at all participating institutions provided approval. The study was registered at ClinicalTrials.gov: NCT03208725.

### Data Collection and Study Variables

Clinical definitions were harmonized across sites through training, standard operating procedures, and case report forms (https://chainnetwork.org/resources/). Demographics, history, prior hospitalization, prior antimicrobials, clinical presentation, and laboratory tests were recorded at admission (Supplementary Table 1). We previously found that pre-admission antimicrobial usage is frequently inaccurately reported, so this data point was collected as Y/N. During admission, daily clinical features and antimicrobial use were recorded on standardized proforma (Supplementary Appendix 2 and 3).

### Outcomes

We examined the proportions of children and the number of child-days in hospital receiving intravenous (IV) or oral antimicrobials, Access, Watch and Reserve categories, and antimicrobial classes received before admission and during the first 48 hours after admission. For children with sepsis, severe pneumonia, or severe malnutrition (recommended first-line treatment of penicillin or ampicillin plus gentamicin), we examined the use of first-, second-, or third-line antimicrobials according to WHO guidelines. Amoxicillin plus clavulanic acid, azithromycin, amikacin, vancomycin, ceftriaxone, ciprofloxacin, levofloxacin, and cefalexin were regarded as second-line, and ceftazidime, carbapenems, and linezolid as third-line (Supplementary Table 1, Supplementary Appendixes 4 and 5d) [17].

### Statistical Analysis

Baseline characteristics and antimicrobial use were summarized using counts and proportions. If data were missing for ≤3 days, with use of the same antimicrobial before and after the gap, no change was assumed. Gaps >3 days were left unimputed. Rates were calculated per 100 inpatient child-days with 95% CIs. We classified children by primary and underlying diagnoses made at admission and discharge as requiring antimicrobial treatment or not according to WHO guidelines and determined the proportion receiving antimicrobials among those without an indication overall and for the syndromes of diarrhea, malaria, and anemia.

To identify factors associated with Watch antimicrobial use, we used multilevel binomial regression and mixed-effects generalized linear modeling (site as a random intercept), backwards stepwise selection (using exit *P* < .10), and inverse sampling weights based on the profile of all admissions to account for intentional oversampling of malnourished children within CHAIN [18]. Weighted simple proportions were also calculated this way. Model performance was assessed with area under the receiver operating characteristic (ROC) curve using 1000 bootstrap samples. Differences between sites or strata were modeled using logistic regression with inverse weighting as appropriate. Marginal means were derived in the response scale using the *emmeans* R package. For pairwise comparisons between strata or sites, *P* values were adjusted following the Tukey procedure.

### Role of the Funding Source

The funder had no role in the study design, data collection, analyses, interpretation, writing, or decision to publish. C.T., M.N., and N.N. had full access to the data, and C.T., J.L.W., N.S., and J.A.B. were responsible for the decision to publish.

## RESULTS

### Participant Characteristics

**Figure 1.**
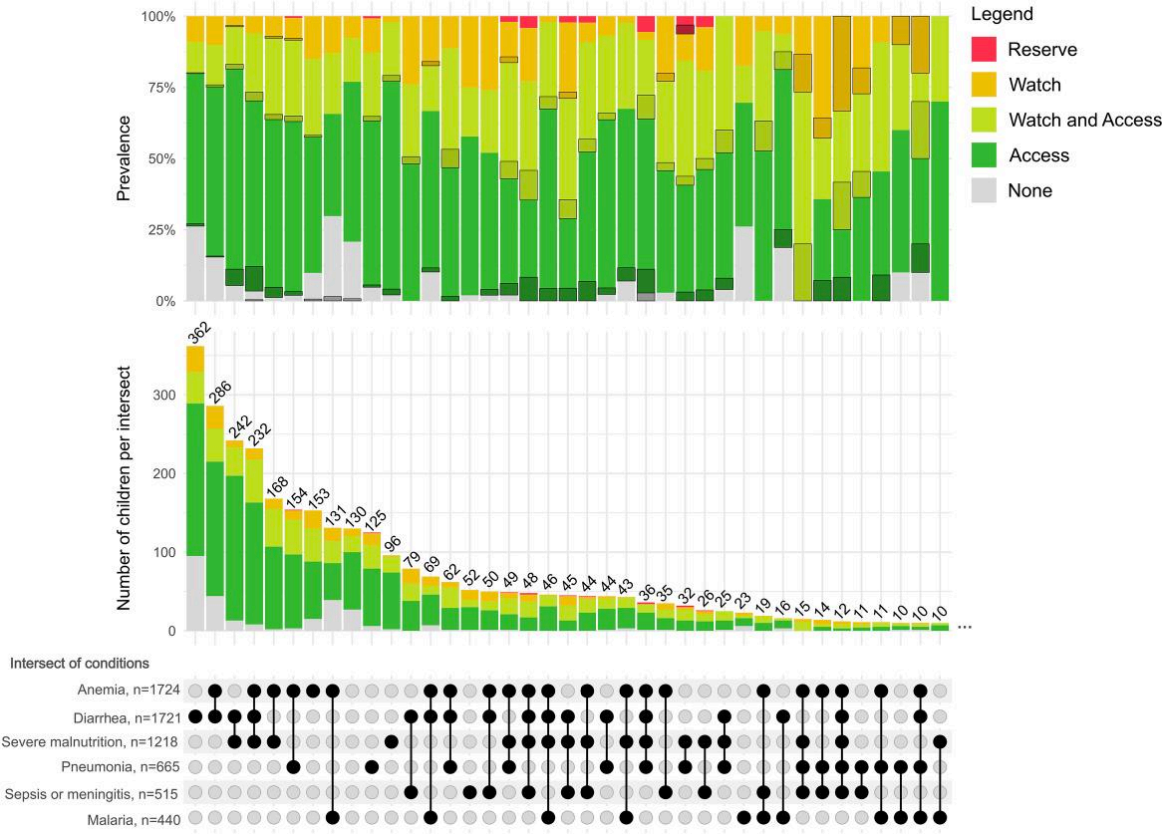
Upset plot detailing antibiotic use in children with specific combinations of most frequent clinical syndromes. The bottom panel represents combinations of clinical syndromes if found in at least 10 patients (x-axis). Combinations of black dots indicate co-presence of conditions. The y-axis in the middle panel shows, for each syndrome combination, the absolute count of children who received the antimicrobial as per the figure legend. The y-axis of the top panel shows the proportion of children who received the different antibiotics for each combination of conditions. The proportion of children who died by syndrome combination and antibiotics received is indicated by grayed shadow boxes.

Overall, 3101 children (median age [interquartile range {IQR}], 11 [7–16] months) were enrolled in 3 nutritional strata: n = 1120 (36%) nonwasted (NW), 763 (25%) moderately wasted (MW), and 1218 (39%) severely wasted or kwashiorkor (edematous malnutrition—SWK) (Table 1, Supplementary Table 1, and Supplementary Figure 1). Frequent diagnoses were anemia (n = 1724 [59%]), diarrhea (n = 1721 [55%]), severe pneumonia (n = 665 [21%]), malaria (n = 419 [21%]), and suspected sepsis (n = 436 [14%]). These conditions co-occurred in 1430 (46%) children, varying by nutritional strata and site (Table 1, Figure 1, and Supplementary Table 1).

**Table 1.** Sociodemographic and Clinical Characteristics of Children by Enrollment Strata

	NW n = 1120, No. (%)	MW n = 763, No. (%)	SWK n = 1218, No. (%)	Overall n = 3101, No. (%)
Demographics				
Sex, female	432 (39)	329 (43)	583 (48)	1344 (43)
Age group				
>6 mo	205 (18)	143 (19)	268 (22)	616 (20)
6–12 mo	405 (36)	316 (41)	432 (35)	1153 (37)
>12 mo	510 (46)	304 (40)	518 (43)	1332 (43)
Site				
Kilifi	117 (10)	52 (6.5)	76 (6.2)	245 (7.9)
Mbagathi	88 (7.9)	81 (11)	110 (9.0)	279 (9.0)
Migori	106 (9.5)	51 (6.7)	123 (10)	280 (9.0)
Kampala	131 (12)	109 (14)	236 (19)	476 (15)
Blantyre	174 (16)	52 (6.8)	107 (8.8)	333 (11)
Karachi	134 (12)	75 (9.8)	140 (11)	349 (11)
Dhaka	125 (11)	109 (14)	160 (13)	394 (13)
Matlab	95 (8.5)	123 (16)	96 (7.9)	314 (10)
Banfora	150 (14)	111 (15)	170 (14)	431 (14)
Clinical presentation at admission				
Pneumonia^a^				
None	695 (62)	519 (68)	905 (74)	2119 (68)
Mild	139 (12)	65 (8.5)	113 (9.3)	317 (10)
Severe	286 (26)	179 (23)	200 (16)	665 (21)
Diarrhea	525 (47)	484 (63)	712 (58)	1721 (55)
Sepsis^b^	142 (13)	96 (13)	198 (16)	436 (14)
Meningitis/encephalopathy^c^	52 (4.6)	24 (3.2)	35 (3.9)	111 (3.6)
Nutritional edema	-	-	353 (29)	353 (11)
Malaria rapid diagnostic test positive	202 (18)	106 (14)	132 (11)	440 (14)
Hemoglobin				
>110 g/dL	216 (20)	116 (16)	196 (17)	528 (18)
100–110 g/dL	265 (25)	166 (23)	252 (22)	683 (23)
70–100 g/dL	443 (42)	310 (43)	530 (46)	1283 (44)
<70 g/dL	135 (13)	121 (17)	185 (16)	441 (15)
Blood glucose				
<3 mmol/L	12 (1.1)	7 (0.9)	32 (2.7)	51 (1.7)
≥3 to ≤10 mmol/L	1022 (93)	702 (94)	1074 (90)	2798 (92)
>10 mmol/L	67 (6.1)	41 (5.5)	85 (7.1)	193 (6.3)
Underlying conditions				
Stunted (LAZ < −2)	269 (24)	348 (46)	925 (76)	1542 (50)
Premature birth or low birth weight^d^	132 (12)	132 (18)	245 (21)	509 (17)
HIV status				
Negative	1028 (92)	692 (91)	1022 (84)	2742 (88)
Exposed	69 (6.2)	46 (6.0)	96 (7.9)	211 (6.8)
Infected	23 (2.1)	25 (3.3)	100 (8.2)	148 (4.8)
Untested	39 (3.5)	14 (1.8)	23 (1.9)	76 (2.5)
Other chronic illness^e^	64 (5.7)	55 (7.2)	98 (8.0)	217 (7.0)
Antimicrobials <7 d before admission	526 (47)	370 (48)	526 (43)	1422 (46)
Prior hospitalization				
<1 wk ago	50 (4.5)	52 (6.8)	73 (6.0)	175 (5.7)
1 wk–1 mo ago	56 (5.0)	42 (5.5)	109 (9.0)	207 (6.7)
>1 mo ago	146 (13)	89 (12)	176 (15)	411 (13)
Outcome				
Time to discharge, d^f^	3.0 (2.0–5.0)	4.0 (2.0–6.0)	7.0 (4.0–12)	4.0 (2.0–7.0)
Died during index admission	22 (2.0)	32 (4.2)	128 (11)	182 (5.9)
Time to inpatient death, d	1.5 (0.0–4.0)	2.0 (1.0–5.3)	3.0 (1.0–8.0)	3.0 (1.0–8.0)
Duration of admission, d	3 (2–5)	4 (2–6)	7 (4–12)	4 (2–7)
Readmitted	173 (15)	129 (17)	204 (17)	506 (16)
Time to death, d	1.5 (0.0–4.0)	2.0 (1.0–5.5)	3.0 (1.0–8.0)	3.0 (1.0–8.0)

Results are presented as frequency (%) or median (IQR).

Abbreviations: AVPU, alert, voice, pain and unresponsive; IQR, interquartile range; LAZ, length for age Z score; MW, moderate wasting; NW, no wasting; SMK, severe wasting or kwashiorkor (severe malnutrition).

^a^Cough or difficulty breathing with oxygen saturation <90%, central cyanosis, or grunting; very severe chest indrawing or inability to breastfeed or drink; or lethargy, reduced level of consciousness, or convulsions.

^b^Clinician diagnosis.

^c^AVPU.

^d^Reported premature (<37 weeks) or low birthweight (<2.5 kg).

^e^Chronic illnesses including thalassemia, cerebral palsy, sickle cell disease, congenital cardiac diseases and known tuberculosis (LAZ).

^f^Index admission for survivors only.

Inpatient case fatality and median (IQR) duration of admission were 182/3101 (5.9%; 95% CI, 5.07–6.75) and 4 (IQR 2–7) days (Table 1). Data for individual clinical syndromes are given in Supplementary Table 2.

### Prior Admission and Antimicrobial Use

Prior hospitalization within the previous month was reported in 382 (11%) children, and within the previous 1–6 months in 411 (13%) children (Table 1). Antimicrobial use <7 days before admission was reported in 1422 (46%) children, varying by site but not by nutritional stratum (Supplementary Figure 2**)**.

### Inpatient Antimicrobial Usage

Daily antimicrobial data were missing for 195/21807 (0.9%) child-days. Overall, antimicrobials were prescribed for 19 398/21 807 (93%) inpatient child-days among 2818/3101 (91%) children. Intravenous antimicrobials were received by 2644/3101 (85%) children. Most (2787/3101 [90%]) children initiated antimicrobials within <48 hours of admission (2608/3101 [84%] for intravenous antimicrobials).

### Types of Antimicrobials

**Figure 2.**
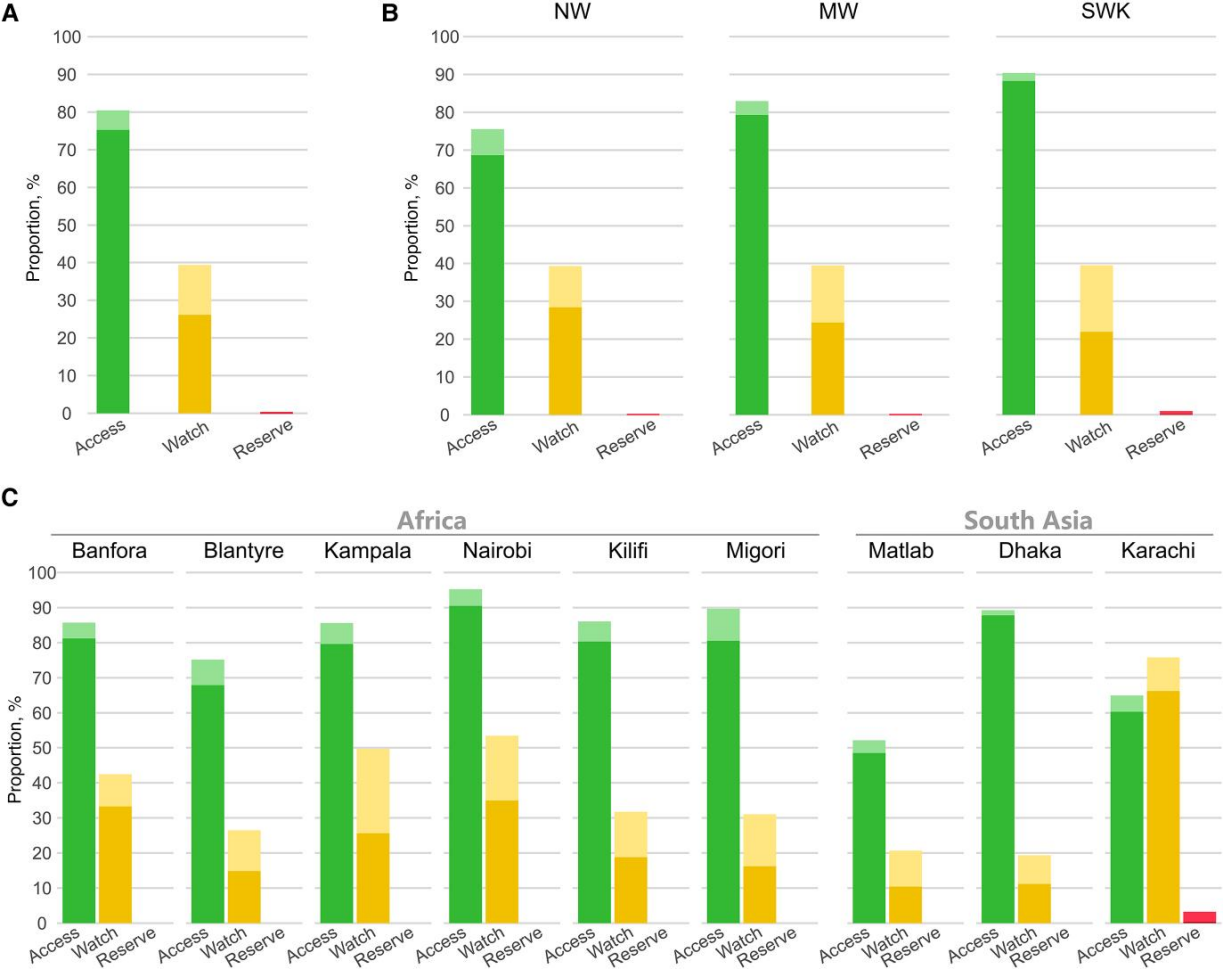
Bar graph representing the percentage of children receiving antimicrobials. Dark-colored bars represent the percentage of children who received antimicrobials within 48 hours of admission, while light-colored bars indicate those starting antimicrobials after 48 hours. The percentage of children who received antimicrobial classes during admission: (A) all children combined, (B) split by nutritional strata, (C) split by split by site. Abbreviations: MW, moderately wasted; NW not wasted; SWK, severely wasted or kwashiorkor (severe malnutrition).

**Figure 3.**
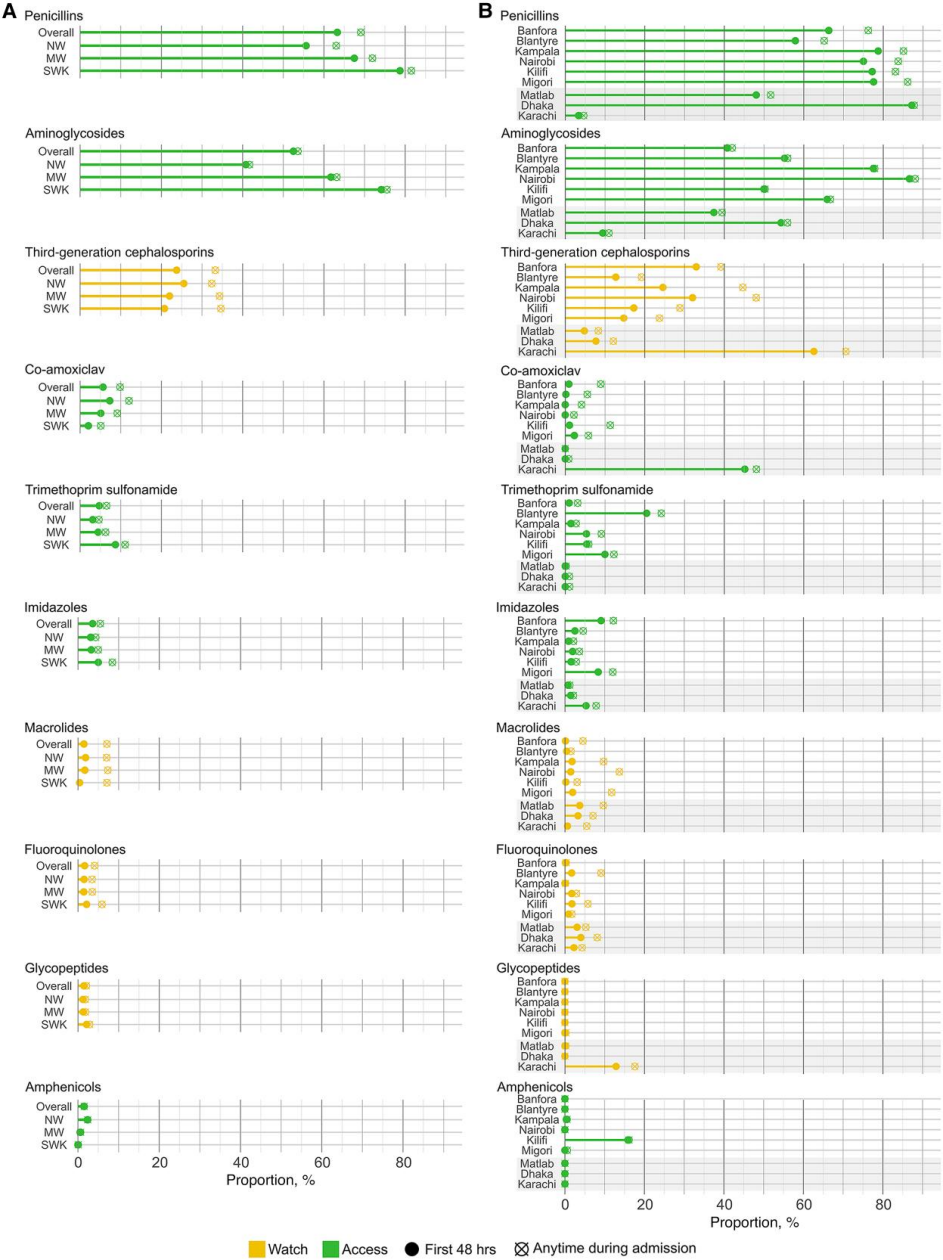
Line graph colors representing percentage of children receiving antimicrobials. A, Split by nutritional strata B, Split by site. Filled dot represents % of children that started antimicrobials within 48 hours of admission while dot with cross bar indicates those starting antimicrobials after 48 hours. Overall percentages weighted by enrollment stratum. Antimicrobial groups not shown if proportions <1%. Abbreviations: NW not wasted; MW, moderately wasted; SWK, severely wasted or kwashiorkor (severe malnutrition).

Overall, 2477 children received (76%) Access, 1092 (35%) Watch, and 12 (0.3%) Reserve antimicrobials (weighted proportions) (Figure 2). Penicillins (n = 2140 [65%]), aminoglycosides (1852 [53%]), and third-generation cephalosporins (990 [31%]) were the most frequent (Figure 3).

Overall, 949/3101 (31%) children received oral antimicrobials (range across sites of 4.6%–62%), with a median (IQR) duration of 1 (0.0–2.0) day during hospitalization (Table 2). Most frequent were oral penicillin (522 [17%]) and co-trimoxazole for HIV prophylaxis (212 [6.8%]) (Table 2).

**Table 2.** Antibiotics Received by Children at Any Time During Admission

Characteristic	NW n = 1120, No. (%)	MW n = 763, No. (%)	SWK n = 1218, No. (%)	Overall n = 3101, No. (%)	Weighted Overall Proportion, %
Mode of administration					
Receiving IV antibiotics	827 (74)	647 (85)	1170 (96)	2644 (85)	81
Days on IV antibiotics	4.0 (0.00–8.0)	6.0 (3.0–11)	10 (6.0–16)	7.0 (3.0–12)	
Receiving oral antibiotics	286 (26)	190 (25)	473 (39)	949 (31)	29
Days on oral antibiotics	1.0 (0.00–2.0)	1.0 (0.00–2.0)	1.0 (0.00–4.0)	1.0 (0.00–2.0)	
Receiving both oral and IV antibiotics	552 (49)	432 (57)	697 (57)	1681 (54)	52
WHO AWaRe classification					
Access	778 (69)	611 (80)	1088 (89)	2477 (80)	76
Watch	373 (33)	267 (35)	452 (37)	1092 (35)	35
Reserve	1 (<0.1)	1 (0.1)	10 (0.8)	12 (0.4)	0.3
First-, second-, and third-line regimens for sepsis^a^					
First-line	700 (63)	569 (75)	1038 (85)	2307 (74)	70
Second-line	429 (38)	293 (38)	469 (39)	1191 (38)	38
Third-line	13 (1.2)	9 (1.2)	40 (3.3)	62 (2.0)	1.7
Antimicrobial line, IV					
First-line IV	580 (52)	520 (68)	1011 (83)	2111 (68)	59
Second-line IV	386 (34)	272 (36)	427 (35)	1085 (35)	35
Third-line IV	12 (1.1)	7 (0.9)	32 (2.6)	51 (1.6)	1.7
Antimicrobial classes					
Penicillins	636 (57)	525 (69)	979 (80)	2140 (69)	65
Aminoglycosides	460 (41)	477 (63)	915 (75)	1852 (60)	53
Penicillins and aminoglycosides	439 (58)	403 (36)	838 (69)	1680 (54)	55
Co-amoxiclav	89 (7.9)	47 (6.2)	49 (4.0)	185 (6.0)	6.7
Imidazoles	39 (3.5)	32 (4.2)	101 (8.3)	172 (5.5)	4.8
Amphenicols	26 (2.3)	5 (0.7)	1 (<0.1)	32 (1.0)	1.5
Antipseudomonal beta-lactams^b^	6 (0.5)	2 (0.3)	17 (1.4)	25 (0.8)	0.7
Lincosamides	0 (0)	0 (0)	1 (<0.1)	1 (<0.1)	<0.1
Macrolides	41 (3.7)	35 (4.6)	66 (5.4)	142 (4.6)	4.3
Fluoroquinolones	22 (2.0)	15 (2.0)	54 (4.4)	91 (2.9)	2.6
Glycopeptides	6 (0.5)	2 (0.3)	17 (1.4)	25 (0.8)	2.1
Third-generation cephalosporins	334 (30)	243 (32)	413 (34)	990 (32)	31
Carbapenems	4 (0.4)	2 (0.3)	10 (0.8)	16 (0.5)	0.5
Oxazolidinones	1 (<0.1)	1 (0.1)	10 (0.8)	12 (0.4)	0.3
Polymyxins	1 (<0.1)	0 (0)	0 (0)	1 (<0.1)	<0.1
Trimethoprim/sulfamethoxazole (HIV prophylaxis)	39 (3.5)	42 (5.5)	131 (11)	212 (6.8)	5.6

Results presented as frequency (%) or median (IQR) in days. Proportions for AWaRe categories and antimicrobial classes total >100% because 2831/3101 (91%) children received >1 type of antibiotic.

Abbreviations: AWaRe, Access, Watch, and Reserve; IQR, interquartile range; IV, intravenous; WHO, World Health Organization.

^a^First-, second-, and third-line agents for sepsis are defined as per WHO guidelines as defined in Supplementary Table 1. The WHO also recommends these agents for children with severe pneumonia and severe malnutrition. Here they are applied to data from all children. Sepsis regimens were also used for severe pneumonia and severe malnutrition.

^b^Beta-lactam antipseudomonal antimicrobials include ampicillin/clavulanic acid or sulbactam and piperacillin/tazobactam (Supplementary Appendix 4).

Overall, 2644 /3101 (85%) children received intravenous antimicrobials (74% of NW, 85% of MW, and 96% of SWK), for a median (IQR) duration of 3 (2.0–3) days. Penicillins (1974 [64%]), aminoglycosides (1852 [60%]), and ceftriaxone (970 [31%]) were the most frequent (Table 2).

### Access Antimicrobials

**Figure 4.**
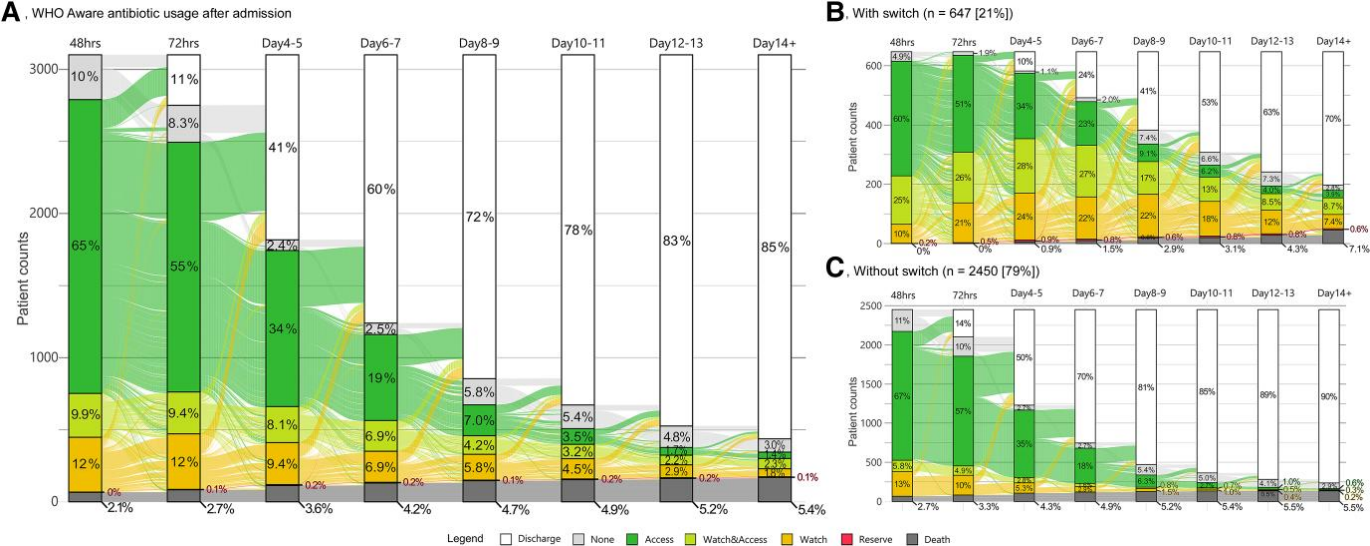
Alluvial plots detailing the proportion of AWaRe antimicrobial use in children during admission presented as sequential 48-hour periods starting from admission. Antimicrobial classes used in (A) all admissions, (B) children receiving antimicrobial changes (switch), and (B) children with no change in antimicrobials. Each alluvial line depicts the progression of a child from admission to discharge (white) or death (gray) and the antimicrobials received, color-coded as per the legend. The y-axis presents stacked child counts. Abbreviation: AWaRe, Access, Watch and Reserve.

Access antimicrobials were used for 62 (95% CI, 61‒63) days per 100 child-days (38 to 81 across sites) (Table 3). Most Access antimicrobials (1875/2884 [65%] were prescribed within 48 hours of admission (Figure 4). A penicillin/aminoglycoside combination was used in 1680/3101 (54%) children (1680/2425 [69%] children who started antimicrobials within 48 hours) (Table 2). Use of this combination ranged from <0.1% in Karachi to 84% in Kampala (Supplementary Table 3) and was more common in children in the SWK stratum (69%; 95% CI, 66%–71%) than the MW (58%; 95% CI, 54%–61%) or SWK (36%; 95% CI, 33%–39%) strata (both *P* < .0001) (Figure 3A). One site, Kilifi, prescribed chloramphenicol in 16% (95% CI, 10%–22%) of children for suspected meningitis with almost no use at other sites.

**Table 3.** Child-Days Spent on Each WHO AWaRe Classification by Site

AWaRe Antimicrobial Classification	No. of Children Receiving This Antimicrobial Classification for ≥1 Day (%)	Total Inpatient Child-Days Receiving This Antimicrobial Classification	Total Inpatient Child-Days of Admission	Days Receiving This Antimicrobial Classification per 100 Inpatient Child-Days (95% CI)
Access				
Kilifi	198 (81)	964	1476	65(61‒70)
Mbagathi	262 (94)	1828	2620	70(67‒73)
Migori	237 (85)	1180	1832	64(61‒68)
Kampala	410 (86)	2429	4847	50 (48‒52)
Blantyre	242 (73)	1129	1920	59 (55‒62)
Karachi	210 (60)	862	2251	38 (36‒41)
Dhaka	365 (93)	2062	2759	75 (72‒78)
Matlab	178 (57)	772	1357	57 (53‒61)
Banfora	375 (87)	2215	2745	81 (77‒84)
Total Access	2477 (80)	13 441	21 807	62 (61‒63)
Watch				
Kilifi	79 (32)	454	1476	31(28‒34)
Mbagathi	143 (51)	1008	2620	39 (36‒41)
Migori	71 (25)	334	1832	18(16‒20)
Kampala	170 (36)	811	4847	17 (16‒18)
Blantyre	77 (23)	334	1920	17 (16‒19)
Karachi	264 (76)	1691	2251	75 (72‒79)
Dhaka	72 (18)	422	2759	15(14‒17)
Matlab	42 (13)	157	1357	12 (9.8‒14)
Banfora	174 (40)	685	2745	25 (23‒27)
Total Watch	1092 (35)	5896	21 807	27 (26‒28)
Reserve				
Kilifi	0	0	1476	0
Mbagathi	0	0	2620	0
Migori	0	0	1832	0
Kampala	0	0	4847	0
Blantyre	0	0	1920	0
Karachi	12 (3.4)	61	2251	2.7 (2.1‒3.5)
Dhaka	0	0	2759	0
Matlab	0	0	1357	0
Banfora	0	0	2745	0
Total Reserve	12 (0.4)	61	21 807	0.3 (0.2‒0.4)

Kilifi (n = 245), Mbagathi (n = 279), Migori (n = 280), Kampala (n = 476), Blantyre (n = 333), Karachi (n = 349), Dhaka (n = 394), Matlab (n = 314), Banfora (n = 431).

Abbreviations: AWaRe, Access, Watch and Reserve; WHO, World Health Organization.

### Watch Antimicrobials

Watch antimicrobials were used for 27 (95% CI, 26‒28) days per 100 child-days among 1092 (35%) children (Tables 2 and 3, Figure 2A), predominantly third-generation cephalosporins (Figures 3A, B). In the first 48 hours, 705 (24%) children received Watch antimicrobials. Watch antimicrobial use ranged from 12 to 75 days per 100 child-days (Table 3), with Civil Hospital, Karachi, predominantly initiating co-amoxiclav (an Access agent) for children in the NW and MW strata and SWK children receiving mostly third-generation cephalosporins. Carbapenems were prescribed in 16 (0.5%) children, of whom 10/16 (63%) were in the SWK stratum (Table 2). Across nutritional strata, there was less Watch antimicrobial usage in more severely malnourished groups (Supplementary Table 4): NW, 32 (IQR, 30‒33); MW, 29 (IQR, 28‒31); and SWK, 24 (95% CI, 23‒25) per 100 child-days (Supplementary Table 5). Use of Watch antibiotics across sites ranged from 42/314 (13%) in Matlab to 264/349 (76%) children in Karachi (Supplementary Table 3). Combination use of Access and Watch antibiotics was highest (25%) among children with antibiotic switching during admission (Supplementary Figure 3).

### Reserve Antimicrobials

Reserve antimicrobials were used in 12 (0.4%) children (specifically, 11 linezolid and 1 polymyxin) for 61 inpatient child-days (0.3; 95% CI, 0.2‒0.4; days per 100 inpatient child-days) (Tables 2 and 3). Reserve antimicrobials were only used at Civil Hospital: 2.7 (95% CI, 2.1‒3.5) days per 100 child-days at that hospital. Eleven of the 12 started Reserve antimicrobials >48 hours after admission, and 10/12 were in the SWK stratum (Table 2, Supplementary Tables 4 and 6, Supplementary Figure 3).

### Children With Sepsis, Severe Malnutrition, and Severe Pneumonia

Of 1886 children with sepsis, severe malnutrition, and/or severe pneumonia at admission, 1509 (80%) received first-line antimicrobials at any time: 270 (66%) NW, 201 (77%) MW, and 1038 (85%) SWK (Supplementary Table 6). These were prescribed for 58 days per 100 child-days (Table 3). Penicillins (74%) and aminoglycosides (70%) were the most common (Table 2). Second-line antimicrobials were used in 806/1866 (43%) children (Supplementary Tables 2 and 6) for 29 days per 100 child-days (Table 3), with NW, MW, and SWK receiving 36, 33, and 24 days per 100 child-days, respectively (Supplementary Table 5). The most common second-line antimicrobials were third-generation cephalosporins (36%), beta-lactam/beta-lactamase inhibitor combinations (8.7%), and macrolides (5.4%) (Table 2). Third-line antimicrobials were used in 53/1886 (2.8%) children for 1.8 (95% CI, 1.6‒2.0) days per 100 inpatient child-days (Table 3 and Supplementary Tables 5 and 7), mostly >48 hours after admission (Supplementary Table 6).

### Overuse of Antimicrobials

We examined antimicrobial use in children with diarrhea, malaria, moderate/severe anemia, and other diagnoses not indicating an antimicrobial in the absence of concurrent conditions for which antibiotics are indicated (Table 4). Most children with these diagnoses (68% in malaria, 78% in diarrhea, and 83% in anemia) also had another diagnosis or comorbidities requiring antibiotic treatment. Overall, 509/3101 (16%) admissions had no indication for an antimicrobial; 341/509 (70%) of these individuals received an antimicrobial, which was similar across diagnoses. Thus, 341/3101 (11%) admissions received an antimicrobial without an indication, 117/341 (34%) of whom received a Watch agent, representing 3.8% of all admissions.

**Table 4.** Antimicrobial Use in Diagnoses Not Indicating an Antimicrobial and All Admissions

Children With a Recorded Diagnosis Not Indicating an Antimicrobial	Children Without Another Indication for an Antimicrobial, No.^a^ (%) [Weighted %]	Children Without an Indication for an Antimicrobial who Received an Antimicrobial, No.		% of Children Without an Indication who Received an Antimicrobial % [Weighted %]	% of All Admitted Patients (n = 3101) Without an Indication who Received an Antimicrobial % [Weighted %]
Diarrhea n = 1721	374 (22) [25]	Any antimicrobial	252	67 [61]	8.1 [8.0]
		Access	211	56 [50]	6.8 [6.5]
		Watch	81	22 [21]	2.6 [2.8]
		Reserve	0	0	0
		None	122	33 [39]	3.9 [5.2]
Malaria n = 440	142 (32) [38]	Any antimicrobial	94	66 [64]	3.0 [3.8]
		Access	74	52 [50]	2.4 [3.0]
		Watch	36	25 [24]	1.1 [1.4]
		Reserve	0	0	0
		None	48	34 [36]	1.5 [2.1]
Moderate/severe anemia n = 1827	309 (17) [20]	Any antimicrobial	222	72 [69]	7.2 [7.7]
		Access	182	59 [55]	5.9 [6.2]
		Watch	85	28 [27]	2.7 [3.1]
		Reserve	0	0	0
		None	87	28 [31]	2.8 [3.5]
Other diagnoses not indicating an antimicrobial n = 433^a^	37 (8.5) [8.6]	Any antimicrobial	27	73 [69]	0.87 [0.97]
		Access	22	59 [53]	0.71 [0.74]
		Watch	8	22 [23]	0.26 [0.32]
		Reserve	0	0	0
		None	10	27 [31]	0.32 [0.43]
All admissions n = 3101	509 (16)^b^ [19]	Any antimicrobial	341	67 [62]	11 [12]
		Access	280	55 [50]	9.0 [9.2]
		Watch	117	23 [22]	3.8 [4.2]
		Reserve	0	0	0
		None	168	33 [38]	5.4 [7.0]

^a^Including allergy/anaphylaxis/atopy, asthma, birth asphyxia/cerebral palsy/developmental delay, congenital abnormality, electrolyte abnormality, failure to thrive, gastroesophageal reflux disease, laryngomalacia, microcephaly, moderate malnutrition, noninfectious dermatosis, rickets, varicella, viral hepatitis.

^b^Children may have had >1 of these diagnoses and/or diagnoses indicating antimicrobials; therefore, the total among all admissions is less than the sum of the rows above.

### Factors Associated With the Use of Watch and Second-Line Antimicrobials

Prior hospital admission, chronic illness, diagnoses of sepsis or meningitis/encephalopathy, blood glucose <3 mmol/L, and duration of admission ≥5 days were positively associated with use of Watch antimicrobials (Table 5). Use of Watch antimicrobials was not independently associated with nutritional strata, use of antimicrobials before admission, WHO danger signs, HIV status, or white blood cell count at admission. Among children with sepsis, severe malnutrition, or severe pneumonia, factors associated with use of second-line antimicrobials were similar (Supplementary Table 8).

**Table 5.** Factors Associated With Receiving Watch Antimicrobials During Admission

		Univariate Analysis		Multivariable Analysis	
Admission Characteristics	Children who Received Watch Antimicrobials (n = 1092), No. (%)	Crude Risk Ratios	*P* Value	Adjusted Risk Ratios	*P* Value
Sex, female	480 (44)	0.98 (0.75‒1.27)	.86	^a^	
Age, mo (log)	-	1.08 (0.93‒1.26)	.32	^a^	
Enrollment strata					
NW	373 (34)	Reference		^a^	
MW	267 (25)	1.12 (0.82‒1.53)	.49	^a^	
SWK	452 (41)	1.15 (0.78‒1.69)	.49	^a^	
Residence				^a^	
Rural	209 (19)	Reference		^a^	
Peri-urban	159 (15)	1.28 (0.96‒1.70)	.09	^a^	
Urban	724 (66)	1.01 (0.57‒1.79)	.96	^a^	
Tertiary hospital level	583 (53)	1.34 (0.43‒4.16)	.62	^a^	
Prior hospital admission	341 (31)	1.59 (1.25‒2.04)	<.001	1.48 (1.14‒1.93)	.003
Antimicrobial <7 d before admission	514 (47)	1.32 (1.18‒1.49)	<.001	^a^	
Stunted (LAZ < −2)	553 (51)	1.08 (0.84‒1.38)	.56	^a^	
HIV status					
Negative	959 (88)	Reference		^a^	
Exposed	54 (4.9)	1.12 (0.66‒1.92)	.67	^a^	
Infected	43 (3.9)	1.75 (1.24‒2.46)	.002	^a^	
Untested	36 (3.3)	1.68 (0.77‒3.68)	.19	^a^	
Features at admission					
Diarrhea	550 (50)	1.11 (0.65‒1.89)	.70	^a^	
Sepsis^b^	224 (21)	2.52 (1.70‒3.74)	<.001	2.64 (1.83‒3.80)	.001
Meningitis/encephalopathy^b^	95 (8.7)	14.1 (5.71‒34.7)	<.001	14.2 (4.75‒42.5)	<.001
Severe pneumonia	317 (29)	1.08 (0.62‒1.88)	.78	1.22 (0.77‒1.93)	.41
Other chronic illness^c^	133 (12)	2.07 (1.21‒3.54)	.008	1.97 (1.10‒3.51)	.02
WHO danger signs^d^	810 (74)	1.30 (0.92‒1.84)	.14	1.22 (0.90‒1.66)	.20
Malaria rapid diagnostic test					
Negative	911 (83)	Reference		^a^	
Positive	162 (14)	1.01 (0.83‒1.22)	.94	^a^	
Not done	19 (1.7)	1.96 (0.64‒5.99)	.24	^a^	
Hemoglobin					
>110 g/dL	182 (17)	Reference		Reference	
100–110 g/dL	205 (19)	0.95 (0.77‒1.17)	.62	0.88 (0.70‒1.09)	.25
70–100 g/dL	523 (48)	1.13 (0.93‒1.37)	.23	0.98 (0.78‒1.22)	.83
<70 g/dL	182 (17)	1.11 (0.88‒1.41)	.38	0.91 (0.70‒1.19)	.49
Blood glucose					
<3 mmol/L	28 (2.6)	2.40 (1.44‒4.01)	.001	2.17 (1.21‒3.88)	.009
3 to 10 mmol/L	1000 (92)	Reference		Reference	
>10 mmol/L	64 (5.9)	0.86 (0.57‒1.30)	.46	0.70 (0.45‒1.09)	.11
WBC					
<5×10^9^/L	30 (2.8)	1.86 (0.97‒3.56)	.06	^a^	
5–17.5×10^9^/L	723 (66)	Reference		^a^	
>17.5×10^9^/L	339 (31)	1.18 (0.88‒1.58)	.27	^a^	
Duration of admission					
<5 d	407 (37)	Reference		Reference	
≥5 d	685 (63)	3.06 (1.82‒5.15)	<.001	3.11 (1.82‒5.32)	<.001

Results presented as frequency (%) or risk ratios.

Abbreviations: AUC, area under the curve; LAZ, length for age Z score; MW, moderate wasting; NW, no wasting; SWK, severe wasting or kwashiorkor (severe malnutrition); WBC, white blood cell count; WHO, World Health Organization.

^a^Variables not selected for inclusion in the multivariable model using backward stepwise selection: NW, MW, SWK. Risk ratio from multilevel mixed-effects generalized linear model with site as random intercept and sample weights, multivariable AUC, 0.78 (95% CI, 0.76‒0.80).

^b^Clinician diagnosis.

^c^Chronic illnesses including thalassemia, cerebral palsy, sickle cell disease, congenital cardiac diseases, and known tuberculosis (LAZ).

^d^Presence of any WHO danger signs: obstructed breathing, respiratory distress, cyanosis, shock, severe anemia, convulsions, severe dehydration, profuse watery diarrhea, vomiting everything, impaired consciousness, temperature >38°C in the last 24 hours or <36°C in last 24 hours (LAZ).

## DISCUSSION

In this prospective cohort in Sub-Saharan Africa and South Asia, 91% of children admitted with acute illness received antimicrobials, including 35% receiving Watch antimicrobials, two-thirds of whom were started within 48 hours of admission.

A previous pediatric point prevalence survey in 56 countries observed substantial variation in use of Access, Watch, and Reserve agents, with ceftriaxone being the most commonly prescribed Watch antimicrobial among hospitalized children in Africa, the Eastern Mediterranean, Europe, and Southeast Asia [5]. The minimal use of Reserve antimicrobials we observed is similar to that survey and an analysis of antibiotic sales in 70 middle- and high-income countries in 2015 [5, 19]. Surprisingly, in the sites involved in these surveys, Access antimicrobials were more often given to neonates than older children, despite their higher mortality risk and duration of exposure to the hospital environment [20].

Known discrepancies between local and published AMR data and current antimicrobial recommendations [21, 22] suggest that coverage of currently recommended Access (most WHO first-line) and some Watch (most WHO second-line) regimens may not be appropriate. Lack of pediatric formulations and sometimes nonavailability or high cost (to health services or families) of carbapenems and Reserve antimicrobials may also limit appropriate use [23].

The susceptibility data available in LMICs to inform empiric prescribing often lack granularity, for example, not distinguishing community from hospital-acquired infections, overrepresenting treatment failures and critical care admissions, or extrapolating data from cultures taken in 1 clinical syndrome or age group to another [1, 24, 25]. In this study, 1 site had the highest use of Watch and Reserve antibiotics, influenced by a national antibiogram, suggesting high rates of resistance to Access and Watch antibiotics [25]. However, such pooled antibiograms commonly include isolates from intensive care–derived infections and adult urinary tract infections, for example, and thus are not necessarily relevant to acutely ill children presenting from the community. Even in settings where microbiological data are more complete, antibiograms can have poor predictive value for individual patient care, and syndrome and context-specific antibiograms are likely to be beneficial [26, 27].

In LMICs, prescribing may be influenced by clinician's choice and patient demand [28]; however, the majority of the children with sepsis, severe malnutrition, and/or severe pneumonia in this study appropriately received first-line antimicrobials at any time during admission as recommended by the WHO. In contrast, two-thirds of first-line ceftriaxone use in Africa has been previously described as inappropriate [29, 30]. We identified clear inappropriate prescribing in children with no indication for antibiotics, with around 70% of patients with uncomplicated gastroenteritis, malaria, or anemia receiving an antimicrobial. However, it is important to consider that overlapping syndromes and comorbidities are frequent [6]. Most children with diagnoses not indicating an antimicrobial had another syndrome or comorbidity indicating antibiotics. This type of overuse represented 11% of admissions, including 3.8% of admissions receiving a Watch agent.

We found limited escalation of antimicrobial therapy among children with severe malnutrition (20% of admissions in these hospitals) [31] and a lack of association of Watch antimicrobial use with WHO danger signs or prehospital antibiotic exposure consistent with treatment failure or resistance selection. This is concerning given that these children may have increased mortality risk and longer stay in the hospital, with vulnerability to hospital-acquired infections [20, 31, 32]. These factors are not included in the current WHO guidelines for antimicrobial selection or timing of antimicrobial escalation and de-escalation. Despite the flexibility within guidelines, they appear to be limiting the appropriate use of Watch and Reserve antimicrobials. For guideline revision, clinical trials testing treatment pathways rather than individual drugs head-to-head are likely to be most informative [18, 24]. Before revision of the guidelines, greater awareness is needed that the guidelines only cover limited situations. Given that most treatment is empiric, risk of death due to age, illness severity, or comorbidities should guide use of broader spectrum agents.

The strengths of this study include a wide range of locations and prospective detailed clinical antimicrobial use data. Limitations included a lack of details on prehospital antimicrobials, some missing data, mostly on the weekends (<1% of days), and a lack of data on prescribing appropriateness or reasons for antibiotic switching. Prior training may have resulted in sites more rigidly sticking to guidelines than would otherwise occur. However, the training provided by CHAIN was in line with training and job-aids provided by the hospitals and national programs.

In conclusion, our findings indicate widespread antibiotic use in hospitalized children across diverse settings in Africa and South Asia, with substantial overuse in conditions where antibiotics are not routinely indicated, such as uncomplicated diarrhea, malaria, and anemia. While WHO-recommended first-line antimicrobials were appropriately used in many cases of sepsis, severe malnutrition, and pneumonia, the overall prescribing pattern suggests that antibiotics were likely unnecessary in a significant proportion of patients. Despite this, there may still be cases of underuse of Watch and Reserve antibiotics in children at elevated risk of mortality with resistant infections. In general, there are limited high-quality data on clinical efficacy to inform guidelines for empiric treatment of previously antibiotic-treated admissions, nonresponders, or hospital-acquired infections. Most government-run hospitals in low-resource settings lack capacity for reliable culture and sensitivity testing. Even where capacity exists, many inpatient pediatric deaths occur within 48 hours, before microbiological results are available. We argue that in such settings, empiric prescribing should be based on individual patient risk of mortality and the likelihood of having a resistant infection. For example, severely underweight children with severe pneumonia have a 5-fold increased inpatient mortality compared with nonunderweight children [33]. Such an approach would enable reduction in inappropriate exposure to broad-spectrum antimicrobials in lower-risk groups and timely, targeted initiation or escalation to Watch or Reserve antibiotics in higher-risk groups. The safety, efficacy, and cost-effectiveness of such strategies need to be tested in clinical trials. In the meantime, greater awareness is needed that the guidelines only cover limited situations, meaning that knowledge and consultation to effectively manage infections need to be employed.

## Supplementary Material

ofaf487_Supplementary_Data

## Supplementary Data

Supplementary materials are available at *Open Forum Infectious Diseases* online. Consisting of data provided by the authors to benefit the reader, the posted materials are not copyedited and are the sole responsibility of the authors, so questions or comments should be addressed to the corresponding author.
