# Correction to: Antimicrobial Usage Among Acutely Ill Hospitalized Children Aged 2‒23 Months in Sub-Saharan Africa and South Asia

**DOI:** 10.1093/ofid/ofag064

**Published:** 2026-02-11

This is a **correction** to:

Caroline Tigoi, Celine Bourdon, Moses Ngari, Robert Musyimi, Molly Timbwa, Shalton Mwaringa, Narshion Ngao, Christopher Maronga, Moses Mburu, Agnes Ndirangu, Fehmina Arif, Zaubina Kazi, Muzammil Shabana Ejaz, Ali Faisal Saleem, Benson O Singa, Ezekiel Mupere, Abu Sadat Mohammad Sayeem Bin Shahid, Al Fazal Khan, Mohammod Jobayer Chisti, Tahmeed Ahmed, Christina Lancioni, Abdoulaye Diallo, Wieger Voskuijl, Robert H Bandsma, Kirkby D Tickell, Priya Sukhtanar, Judd L Walson, Nicole Stoesser, James A Berkley, Antimicrobial Usage Among Acutely Ill Hospitalized Children Aged 2‒23 Months in Sub-Saharan Africa and South Asia, *Open Forum Infectious Diseases*, Volume 12, Issue 9, September 2025, ofaf487, https://doi.org/10.1093/ofid/ofaf487

In the originally published version of this manuscript, author, Caroline Tigoi’s, name was misspelled.

This error has been corrected.

