# Supplementary material for: Antimicrobial Usage Among Acutely Ill Hospitalized Children Aged 2‒23 Months in Sub-Saharan Africa and South Asia
Source: Open Forum Infect Dis. 2025 Aug 18;12(9):ofaf487. doi: 10.1093/ofid/ofaf487 (PMC12448463; doi:10.1093/ofid/ofaf487)
Supplement: ofaf487_Supplementary_Data [file ofaf487_supplementary_data.docx]

*Supplementary Materials*

**Antimicrobial usage among acutely ill hospitalised children aged 2-23 months in sub-Saharan Africa and South Asia.**

The Childhood Acute Illness and Nutrition (CHAIN) Network

Table of Contents

[1. Ethical approvals 3](#_Toc197964415)

[2. Supplementary Methods 3](#_Toc197964416)

[2.1 Participating Sites 3](#_Toc197964417)

[2.2 Exposures 3](#_Toc197964418)

[2.3 Clinical variables 4](#_Toc197964419)

[3. Data management 4](#_Toc197964420)

[3.1 Data cleaning 4](#_Toc197964421)

[3.2 Post-study data cleaning procedures and treatment of missing data 5](#_Toc197964422)

[4. Supplemental Results 5](#_Toc197964423)

[4.1 Supplementary Figures 5](#_Toc197964424)

[Supplementary Figure S1. Tally of participants recruited at each site split by nutritional strata at enrolment 5](#_Toc197964425)

[Supplementary Figure S2. Percent and number of children reported to have received antibiotics in the 7 days prior to hospital admission 6](#_Toc197964426)

[Supplementary Figure S3. Alluvial plots detailing the proportion of AWARE antibiotic use at each site in children during admission presented for sequential 48-hour periods starting from admission 7](#_Toc197964427)

[4.2 Supplementary tables 8](#_Toc197964428)

[Supplementary Table S1: Participant Characteristics 8](#_Toc197964429)

[Supplementary Table S2: Multiple diagnosis table 10](#_Toc197964430)

[Supplementary Table S3: Distribution of antibiotics prescribed for children admitted stratified by site at any time during their admission. 11](#_Toc197964431)

[Supplementary Table S4: Antimicrobials classes prescribed within 48 hours of admission by cohort nutritional status strata. 13](#_Toc197964432)

[Supplementary Table S5: Antimicrobials by cohort nutritional status strata 14](#_Toc197964433)

[Supplementary Table S6: Antibiotic prescriptions for 1886 children with antibiotic syndromes (sepsis, severe pneumonia, and severe malnutrition) 15](#_Toc197964434)

[Supplementary Table S7: Antimicrobials received by hospital site. 16](#_Toc197964435)

[Supplementary Table S8: Factors associated with receiving Second-line antimicrobials. 18](#_Toc197964436)

[5. Appendix 2-Daily review CRF 21](#_Toc197964437)

[6. Appendix 3-Initial treatment CRF 23](#_Toc197964438)

[7. Antimicrobial classification matrix 24](#_Toc197964439)

[8. Appendix 4: WHO treatment guidelines for sepsis and Meningitis as per 2013 WHO Pocketbook of hospital care for children: guidelines for the management of common childhood illnesses. 25](#_Toc197964440)

[9. Appendix 5: Statistical Analysis Plan 28](#_Toc197964441)

# Ethical approvals

The study was approved by the listed ethical committees representing all recruiting and coordinating countries:

1. UK: Oxford Tropical Research Ethics Committee
2. Kenya: Scientific & Ethical Review Unit (SERU), Kenya Medical Research Institute
3. USA: University of Washington Institutional Review Board; Oregon Health and Science University Institutional Review Board
4. Uganda: Makerere University School of Biomedical Sciences Research Ethics Committee
5. Pakistan: Ethical Review Board, Aga Khan University
6. Bangladesh: International Centre for Diarrheal Disease Research: Research Review Committee (RRC) and Ethical Review Committee (ERC)
7. Malawi: COMREC, Kamuzu University of Health Sciences, Malawi
8. Burkina Faso: Comité d’éthique institutionnel du Centre MURAZ
9. Canada: Research Ethics Board of the Hospital for Sick Children
10. The Netherlands Medical Ethics Review Committee, Amsterdam UMC, The Netherlands

# Supplementary Methods

## Participating Sites

Infants were enrolled from nine hospitals in six countries across Africa and South Asia including four rural sites: Matlab hospital in Bangladesh, Kilifi County Hospital in Kenya, Migori sub-county hospital in Kenya and Banfora regional hospital in Burkina Faso.

The CHAIN network recruited at six African sites (Burkina Faso: Banfora Referral Hospital, Kenya: Kilifi County Hospital; Mbagathi Sub-County Hospital, Nairobi; Migori County Hospital, Malawi: Queen Elizabeth Hospital, Blantyre, and Uganda: Mulago Hospital, Kampala).

All sites serve vulnerable populations and represent a range of environments, populations, access to health care, and have differing levels of background comorbidities such as malaria and HIV.

## Exposures

Exposure variables were collected using standardized Case Report Form (CRF)s together with detailed study standard operation procedure (SOP)s which guided staff training and research activities. These included clinical data collection during hospitalization for all the children including demographics, daily reviews, clinical features and diagnosis, progress and treatment received.

Supplementary Table S1. Description of exposures variable type and categorisation.

| **Exposure** | **Variable type and categorisation** |
| --- | --- |
| 1. Age | Continuous |
| 1. Gender | M/F |
| 1. Anthropometry | NW, MW, SWK and Community |
| 1. Previously admitted to hospital | - < 1 week ago - 1 week-1 month ago - >1 month ago |
| 1. Medication in the last 7 days | Y/N (as caregiver reporting of specific antibiotics was unreliable) |
| 1. Initial diagnosis (include those > 5%) | Infection – sepsis, pneumonia, malaria, gastroenteritis, skin and soft tissue infections, bronchiolitis, suspected meningitis, otitis media, febrile convulsions, unknown febrile illness, enteric fever and UTI |
| 1. Chronic conditions | Pulmonary TB, HIV, Sickle Cell Disease, cerebral palsy, neurological problems/epilepsy, renal impairment, congenital cardiac disease |
| 1. Illness severity | Low, Medium or High score as per the main CHAIN cohort analysis published in Diallo et al. 2022. |
| 1. Available laboratory results | Blood culture – positive/negative/ unavailable   - Unavailable results - Abnormal WBC (high/low) - Malaria RDT |
| 1. Level of facility | National referral hospital, regional hospital or district/sub-regional level hospital. |
| 1. Rural and urban | R/U |
| 1. Local antibiotic guidelines | Y/N (1^st^ line – Amp-Gent or Pen-Gentamicin or Benzylpenicillin, or ceftriaxone/cefuroxime if meningitis is suspected) |

## Clinical variables

Description of clinical variables used:

- **WHO AWaRe antibiotic classification** (Access, Watch or Reserve) and WHO prescribing indicators which include the average number of drugs prescribed per encounter, the percentage of drugs prescribed by generic name, the percentage of encounters where an antibiotic was prescribed, the percentage of encounters where an injection was the route of administration and the percentage of drugs prescribed from the Essential Drugs List (EDL) or another recognized formulary will be constructed.
- **SIRS – Systemic Inflammatory Response Syndrome** was defined in accordance with the International Consensus Conference on Pediatric Sepsis, and includes: The presence of at least two of the following four criteria; heart rate low (<90) or high (>180)/min; temperature low(<36^○^C) or high (≥38.5^○^C); respiratory rate high (>34 breaths per minute) and WBC low (<5 x 10^9^/l) or high (>17.5 x 10^9^/l).
- **Severe pneumonia –** Defined by using the WHO (2013) guideline; cough/difficulty breathing with either central cyanosis or oxygen saturation <90% or lower chest wall indrawing or inability to drink/breast fed/vomiting everything or impaired consciousness.
- **Diarrhea –** Defined by using the WHO (2013) guideline; passage of at least three loose or watery stools in a 24 hrs period.
- **Hypoglycemia and hyperglycemia** were defined as blood glucose <3 mmol/L and >10 mmol/L respectively.
- **Malaria –** Defined as positive rapid Malaria test (CareStart HRP2/pLDH).
- **Anaemia –** Defined following WHO guidelines: none (hemoglobin >11 g/dl), mild (hemoglobin ≥10 to 11 g/dl), moderate (hemoglobin ≥7 to <10 g/dl) and severe (hemoglobin <7g/dl)
- **Comorbid/chronic conditions** including TB, HIV, sickle cell among others as binary variables.
- **Laboratory results** like blood culture and white cell count were classified as positive/Negative/unavailable for blood culture or/and for white blood cell count: normal, 6-14; abnormal, <1 or >30; low, <6 or high, >25.
- **Clinical deterioration** after 48 hours of admission based on new onset of any WHO danger signs (Yes/No) i.e., Obstructed breathing, Respiratory distress, Cyanosis, Shock, Severe Anaemia, Convulsions, Severe dehydration, Profuse watery Diarrhoea, Vomiting everything, Impaired consciousness, Temperature >38^○^C in last 24hrs and Temperature >36^○^C in last 24 hrs.

# Data management

## Data cleaning

Throughout the study, collected data underwent ongoing cleaning by a central data management team utilising a dashboard to flag and visualise queries. Dashboards were updated every 30 minutes and issues were circled back to sites for resolution. A data curation sprint was held every two to three weeks to assist the analysis and site-level query resolution process, culminating in the final data being locked and released for analysis. Data curation was started to support preliminary analyses as they became due. Cleaning standard procedures were developed, and the statistical analysis plan was followed for final cleaning.

## Post-study data cleaning procedures and treatment of missing data

To probe for issues, continuous variables were mapped and compared over time. Outliers, discrepancies, and missing data were queried. Categorical variables were examined for consistency and associated clinical and laboratory data were correlated.

A total of 19,907 daily reviews were collected and examined for the analysis of antimicrobial use. First, obvious errors in dates were corrected based on the expected sequence between daily reviews. Then, 440 records were excluded since indicated as incomplete and all records related to readmissions were removed. Free text entries of antibiotic names reported to be given were cleaned and reclassified as appropriate. When antimicrobial entries were missing, gaps of 3 days or less were filled if the antimicrobials were not changed between entries before and after the gap. Periods longer than three days were not imputed. Missing clinical or routine lab test results were assumed to be missing at random indicated as not done like HIV, blood glucose and Malaria RDT.

# Supplemental Results

## Supplementary Figures

**
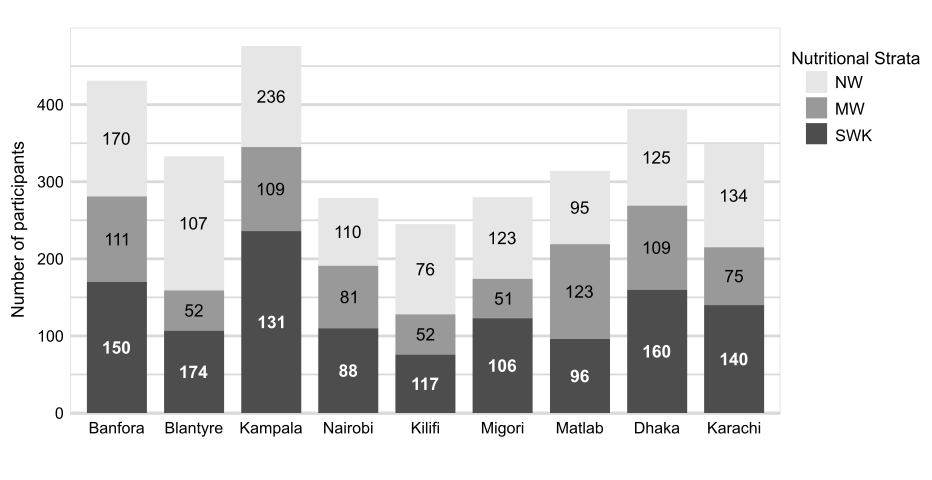
**

Supplementary Figure S1. Tally of participants recruited at each site split by nutritional strata at enrolment. Numbers within bars indicate participant counts per sub-category as per legend. NW, not wasted; MW, moderate wasting; SWK, severe wasting or kwashiorkor.

**
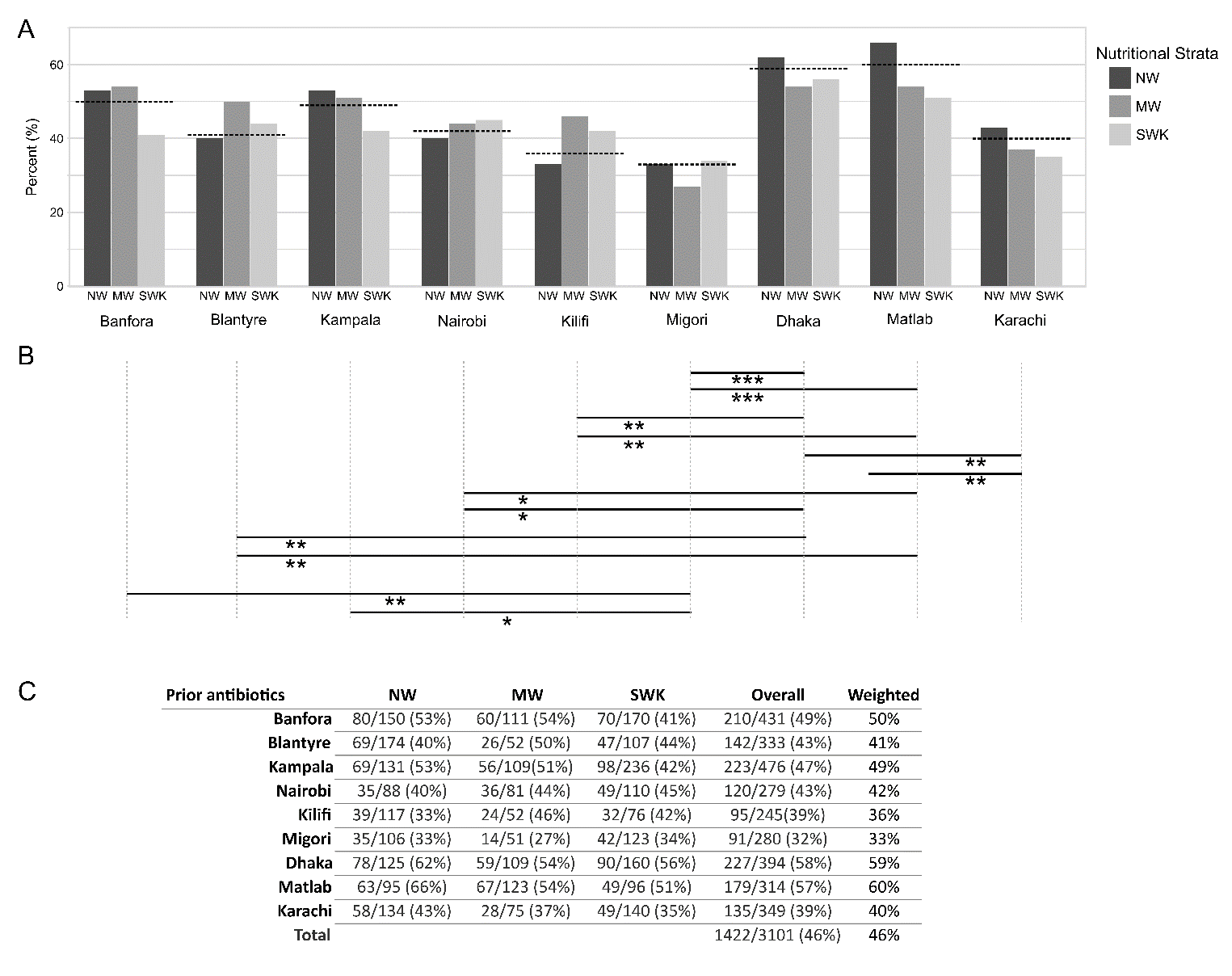
**

Supplementary Figure S2. Percent and number of children reported to have received antibiotics in the 7 days prior to hospital admission. A) Bar chart presenting percentages split by site and nutritional strata. Dashed black line indicates the weighted overall average for each site; B) Line graph indicating significant differences between site as obtained from pairwise comparisons derived from logistic regression models, stars indicated significance threshold as follows: *p<0.05; **p<0.001, ***p<0.0001; C) Table detailing counts and percentages including unweighted- and weighted- overall values. NW, not wasting; MW, moderate wasting; SWK, severe wasting or kwashiorkor.

**
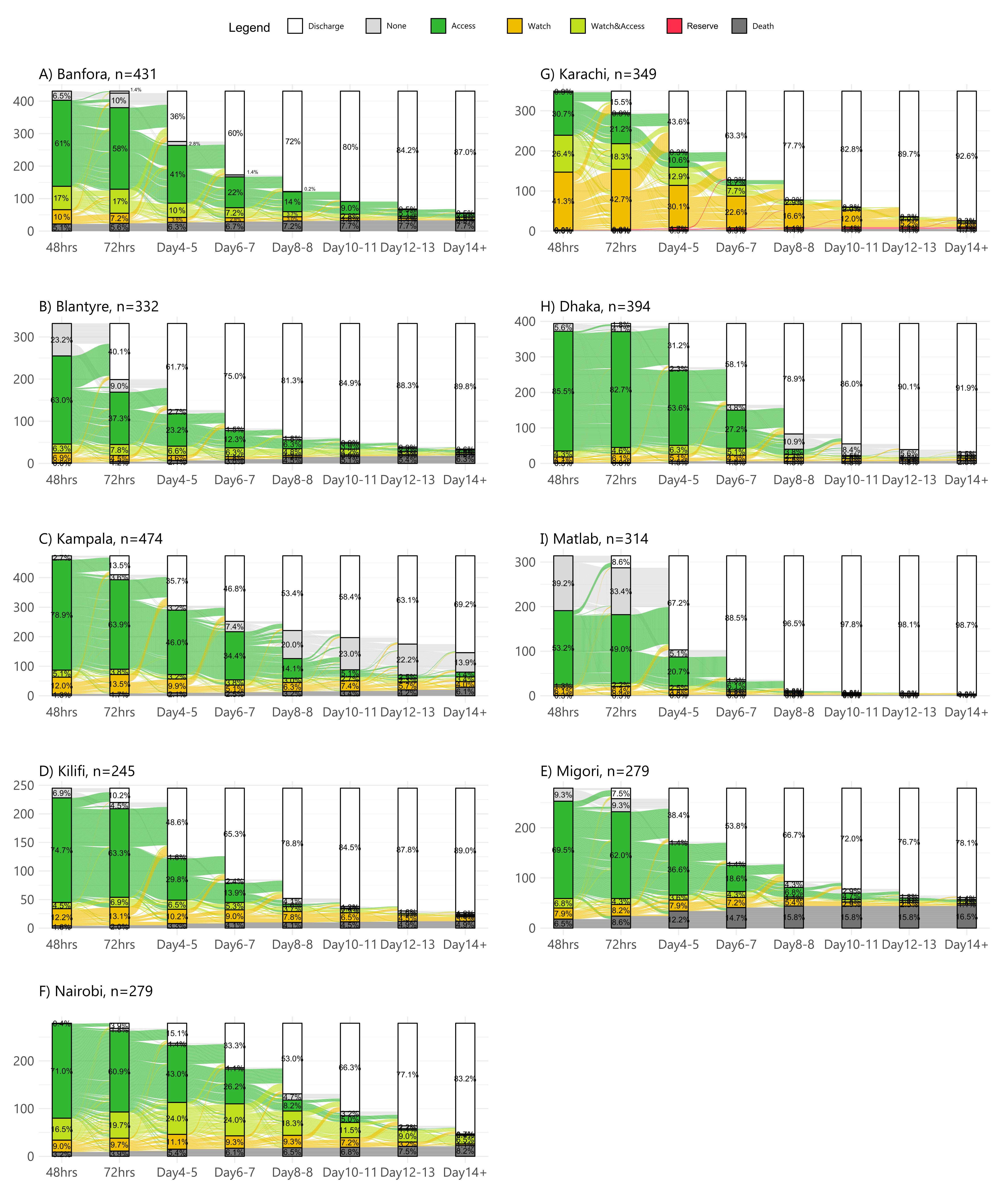
**

Supplementary Figure S3. Alluvial plots detailing the proportion of AWARE antibiotic use at each site in children during admission presented for sequential 48-hour periods starting from admission. Antibiotic class used in A) All admissions; B) children for whom antibiotic prescription changes (switch) and B) children with no change in antibiotic prescription. Each alluvial line depicts the progression of a child through admission to discharge (white) or death (grey) and the antibiotics received colour coded as per legend. Y-axis presents stacked child counts.

## Supplementary tables

## Supplementary Table S1: Participant Characteristics

|  | | | | **Banfora**  **N = 431** | | | **Blantyre N = 333** | | | **KampalaN = 476** | | | **Kilifi**  **N = 245** | | | **Migori**  **N = 280** | | | **Nairobi**  **N = 279** | | | **Karachi**  **N = 349** | | | **Dhaka**  **N = 394** | | | **Matlab**  **N = 314** | | **Overall  N =3,101** |
| --- | --- | --- | --- | --- | --- | --- | --- | --- | --- | --- | --- | --- | --- | --- | --- | --- | --- | --- | --- | --- | --- | --- | --- | --- | --- | --- | --- | --- | --- | --- |
| **Demographics** | | | |  | | |  | | |  | | |  | | |  | | |  | | |  | | |  | | |  | |  |
| Sex, female | | | | 185 (43%) | | | 148 (44%) | | | 215 (45%) | | | 106 (43%) | | | 121 (43%) | | | 128 (46%) | | | 159 (46%) | | | 150 (38%) | | | 132 (42%) | | 1,344 (43%) |
| Age group | | | |  | | |  | | |  | | |  | | |  | | |  | | |  | | |  | | |  | |  |
| *> 6 months* | | | | 66 (15%) | | | 54 (16%) | | | 49 (10%) | | | 46 (19%) | | | 67 (24%) | | | 64 (23%) | | | 106 (30%) | | | 117 (30%) | | | 47 (15%) | | 616 (20%) |
| *6-12 months* | | | | 127 (29%) | | | 113 (34%) | | | 204 (43%) | | | 77 (31%) | | | 87 (31%) | | | 110 (39%) | | | 122 (35%) | | | 166 (42%) | | | 147 (47%) | | 1,153 (37%) |
| *>12 months* | | | | 238 (55%) | | | 166 (50%) | | | 223 (47%) | | | 122 (50%) | | | 126 (45%) | | | 105 (38%) | | | 121 (35%) | | | 111 (28%) | | | 120 (38%) | | 1,332 (43%) |
| **Clinical presentation** | | | |  | | |  | | |  | | |  | | |  | | |  | | |  | | |  | | |  | |  |
| Pneumonia* | | | |  | | |  | | |  | | |  | | |  | | |  | | |  | | |  | | |  | |  |
| *None* | | | | 338 (78%) | | | 247 (74%) | | | 347 (73%) | | | 158 (64%) | | | 195 (70%) | | | 109 (39%) | | | 162 (46%) | | | 270 (69%) | | | 293 (93%) | | 2,119 (68%) |
| *Mild* | | | | 24 (5.6%) | | | 48 (14%) | | | 49 (10%) | | | 9 (3.7%) | | | 53 (19%) | | | 28 (10%) | | | 26 (7.4%) | | | 67 (17%) | | | 13 (4.1%) | | 317 (10%) |
| *Severe* | | | | 69 (16%) | | | 38 (11%) | | | 80 (17%) | | | 78 (32%) | | | 32 (11%) | | | 142 (51%) | | | 161 (46%) | | | 57 (14%) | | | 8 (2.5%) | | 665 (21%) |
| Impaired consciousness, AVPU>A‡ | | | | 45 (10%) | | | 4 (1.2%) | | | 6 (1.3%) | | | 23 (9.4%) | | | 4 (1.4%) | | | 25 (9.0%) | | | 9 (2.6%) | | | 29 (7.4%) | | | 1 (0.3%) | | 146 (4.7%) |
| Diarrhoea | | | | 185 (43%) | | | 148 (44%) | | | 237 (50%) | | | 83 (34%) | | | 146 (52%) | | | 120 (43%) | | | 143 (41%) | | | 382 (97%) | | | 277 (88%) | | 1,721 (55%) |
| Sepsis^†^ | | | | 15 (3.5%) | | | 107 (32%) | | | 180 (38%) | | | 32 (13%) | | | 11 (3.9%) | | | 14 (5.0%) | | | 60 (17%) | | | 14 (3.6%) | | | 3 (1.0%) | | 436 (14%) |
| Nutritional oedema | | | | 36 (8.4%) | | | 34 (10%) | | | 135 (28%) | | | 22 (9.0%) | | | 55 (20%) | | | 13 (4.7%) | | | 28 (8.0%) | | | 19 (4.8%) | | | 11 (3.5%) | | 353 (11%) |
| Malaria rapid diagnostic test positive | | | | 222 (52%) | | | 45 (14%) | | | 38 (8.0%) | | | 32 (13%) | | | 67 (24%) | | | 15 (5.4%) | | | 19 (5.6%) | | | 2 (0.5%) | | | 0 (0%) | | 440 (14%) |
| Haemoglobin g/dl | | | |  | | |  | | |  | | |  | | |  | | |  | | |  | | |  | | |  | |  |
| *>110 g/L* | | | | 26 (6.1%) | | | 77 (29%) | | | 117 (25%) | | | 22 (9.4%) | | | 26 (9.9%) | | | 29 (11%) | | | 53 (15%) | | | 95 (24%) | | | 83 (30%) | | 528 (18%) |
| *100–110 g/L* | | | | 35 (8.2%) | | | 68 (25%) | | | 124 (27%) | | | 54 (23%) | | | 40 (15%) | | | 56 (22%) | | | 79 (23%) | | | 130 (33%) | | | 97 (35%) | | 683 (23%) |
| *70–100 g/L* | | | | 155 (36%) | | | 103 (38%) | | | 184 (40%) | | | 133 (57%) | | | 134 (51%) | | | 141 (55%) | | | 175 (50%) | | | 162 (41%) | | | 96 (34%) | | 1,283 (44%) |
| *<70 g/L* | | | | 209 (49%) | | | 22 (8.1%) | | | 38 (8.2%) | | | 26 (11%) | | | 62 (24%) | | | 31 (12%) | | | 41 (12%) | | | 7 (1.8%) | | | 5 (1.8%) | | 441 (15%) |
| Blood glucose mmol/L | | | |  | | |  | | |  | | |  | | |  | | |  | | |  | | |  | | |  | |  |
| ≥ 3 to ≤ 10 | | | | 399 (93%) | | | 308 (94%) | | | 436 (92%) | | | 223 (92%) | | | 203 (84%) | | | 247 (89%) | | | 321 (94%) | | | 361 (92%) | | | 300 (96%) | | 2,798 (92%) |
| < 3 | | | | 11 (2.6%) | | | 5 (1.5%) | | | 12 (2.5%) | | | 9 (3.7%) | | | 3 (1.2%) | | | 6 (2.2%) | | | 3 (0.9%) | | | 0 (0%) | | | 2 (0.6%) | | 51 (1.7%) |
| > 10 | | | | 21 (4.9%) | | | 15 (4.6%) | | | 25 (5.3%) | | | 11 (4.5%) | | | 35 (15%) | | | 25 (9.0%) | | | 16 (4.7%) | | | 33 (8.4%) | | | 12 (3.8%) | | 193 (6.3%) |
| **Underlying conditions** | | | |  | | |  | | |  | | |  | | |  | | |  | | |  | | |  | | |  | |  |
| Stunting (LAZ <-2) | | | | 167 (39%) | | | 171 (52%) | | | 289 (61%) | | | 97 (40%) | | | 130 (47%) | | | 125 (45%) | | | 186 (53%) | | | 225 (57%) | | | 152 (48%) | | 1,542 (50%) |
| Premature birth or low birth weight^¶^ | | | | 52 (13%) | | | 60 (19%) | | | 81 (18%) | | | 38 (16%) | | | 16 (5.9%) | | | 46 (17%) | | | 27 (7.8%) | | | 89 (23%) | | | 100 (32%) | | 509 (17%) |
| HIV status | | | |  | | |  | | |  | | |  | | |  | | |  | | |  | | |  | | |  | |  |
| *Negative* | | | | 424 (98%) | | | 222 (67%) | | | 379 (80%) | | | 218 (89%) | | | 197 (70%) | | | 249 (89%) | | | 347 (99%) | | | 392 (99%) | | | 314 (100%) | | 2,742 (88%) |
| *Exposed* | | | | 2 (0.5%) | | | 62 (19%) | | | 61 (13%) | | | 10 (4.1%) | | | 52 (19%) | | | 20 (7.2%) | | | 2 (0.6%) | | | 2 (0.5%) | | | 0 (0%) | | 211 (6.8%) |
| *HIV infected* | | | | 5 (1.2%) | | | 49 (15%) | | | 36 (7.6%) | | | 17 (6.9%) | | | 31 (11%) | | | 10 (3.6%) | | | 0 (0%) | | | 0 (0%) | | | 0 (0%) | | 148 (4.8%) |
| Other chronic illness^\|\|^ | | 17 (3.9%) | | | 20 (6.0%) | | | 20 (4.2%) | | | 33 (13%) | | | 6 (2.1%) | | | 13 (4.7%) | | | 101 (29%) | | | 6 (1.5%) | | | 1 (0.3%) | | | 217 (7.0%) | |
| Antimicrobials <7 days prior to admission | | 210 (49%) | | | 142 (43%) | | | 223 (47%) | | | 95 (39%) | | | 91 (33%) | | | 120 (43%) | | | 135 (39%) | | | 227 (58%) | | | 179 (57%) | | | 1,422 (46%) | |
| Prior hospitalization | | | |  | | |  | | |  | | |  | | |  | | |  | | |  | | |  | | |  | |  |
| *< 1 week ago* | | 19 (4.4%) | | | 3 (0.9%) | | | 17 (3.6%) | | | 6 (2.5%) | | | 10 (3.6%) | | | 4 (1.4%) | | | 36 (10%) | | | 30 (7.6%) | | | 50 (16%) | | 175 (5.7%) | |  |
| *1 week-1 month ago* | | 14 (3.2%) | | | 27 (8.1%) | | | 45 (9.6%) | | | 12 (5.0%) | | | 14 (5.0%) | | | 15 (5.4%) | | | 40 (11%) | | | 31 (7.9%) | | | 9 (2.9%) | | 207 (6.7%) | |  |
| *>1 month ago* | | 59 (14%) | | | 68 (20%) | | | 69 (15%) | | | 40 (17%) | | | 13 (4.6%) | | | 40 (14%) | | | 35 (10%) | | | 67 (17%) | | | 20 (6.4%) | | 411 (13%) | |  |
| **Outcome** | | | |  | | |  | | |  | | |  | | |  | | |  | | |  | | |  | | |  | |  |
| Time to discharge, days^**^ | | | | 4.0 (3.0, 7.0) | | | 3.0 (1.0, 6.0) | | | 6.0 (3.0, 15) | | | 3.0 (2.0, 7.0) | | | 4.0 (3.0, 7.0) | | | 7.0 (5.0, 10) | | | 4.0 (2.0, 7.0) | | | 5.0 (3.0, 7.0) | | | 3.0 (2.0,4.0) | | 4.0 (2.0, 7.0) |
| Died during index admission | | | | 33 (7.7%) | | | 21 (6.3%) | | | 29 (6.1%) | | | 12 (4.9%) | | | 47 (17%) | | | 23 (8.2%) | | | 6 (1.7%) | | | 8 (2.0%) | | | 3 (1.0%) | | 182 (5.9%) |
| Time to inpatient death, days | | | | 1.0 (0.00, 2.0) | | | 7.0 (4.0, 9.0) | | | 8.0 (2.0, 12) | | | 2.5 (0.00, 5.8) | | | 2.0 (1.0, 5.0) | | | 3.0 (1.0, 8.5) | | | 5.5 (1.5, 10) | | | 3.0 (1.8, 5.8) | | | 3.0 (2.0, 9.5) | | 3.0 (1.0, 8.0) |
| Readmitted | | | | 45 (10%) | | | 65 (20%) | | | 77 (16%) | | | 40 (16%) | | | 24 (8.6%) | | | 55 (20%) | | | 65 (19%) | | | 94 (24%) | | | 41 (13%) | | 506 (16%) |

Results presented as frequency (%) or median (IQR). * Cough or difficulty breathing with oxygen saturation <90%, central cyanosis, or grunting; very severe chest indrawing or inability to breastfeed or drink; or lethargy, reduced level of consciousness, or convulsions. † Clinician diagnosis. ‡ AVPU, alert, voice, pain and unresponsive; ¶ reported premature (<37 weeks) or low birthweight (<2.5 kg); || Chronic illnesses including thalassemia, cerebral palsy, sickle cell disease, congenital cardiac diseases and known tuberculosis; LAZ: length for age Z score; ** Index admission for survivors only.

## Supplementary Table S2: Multiple diagnosis table

| **Diagnosis 1 →** | **All sepsis cases  (N=436)** | **All Meningitis/ encephalopathy**  **cases (N=104)** | **Severe pneumonia (N=665)** | **Severe malnutrition (N=1407)** | **Malaria  (N=440)** | **Moderate/severe anemia (N=1827)** | **Diarrhea  (N=1811)** | **None of these diagnoses**  **(N=108)** |
| --- | --- | --- | --- | --- | --- | --- | --- | --- |
|  | Any= 433 (99%) | Any= 104(100%) | Any= 649 (98%) | Any= 1372 (98%) | Any= 381 (87%) | Any= 1703 (93%) | Any= 1634 (90%) | Any= 87 (81%) |
|  | Access = 355 (81%) | Access = 57 (55%) | Access = 569(86%) | Access = 1262 (90%) | Access = 333(76%) | Access = 1502 (82%) | Access = 1445(80%) | Access = 79 (73%) |
|  | Watch = 225 (52%) | Watch = 96 (92%) | Watch = 319(48%) | Watch = 515 (37%) | Watch = 165(38%) | Watch = 712 (39%) | Watch = 582 (32%) | Watch = 25 (23%) |
|  | Reserve = 4 (0.9%) | Reserve = 2 (1.9%) | Reserve = 7(1.1%) | Reserve = 10 (0.7%) | Reserve = 0 | Reserve = 7 (0.4%) | Reserve = 5 (0.3%) | Reserve 0 |
| Diagnosis 2 ↓ | Deaths 45 (10%) | Deaths 14 (13%) | Deaths 69 (10%) | Deaths 135 (9.6%) | Deaths 26 (5.9%) | Deaths 140 (7.7%) | Deaths 122 (6.7%) | Deaths 0 |
| **Meningitis/** | N=25 |  |  |  |  |  |  |  |
| **encephalopathy** | Access 13 (52%) |  |  |  |  |  |  |  |
|  | Watch 22 (88%) |  |  |  |  |  |  |  |
|  | Reserve 1 (4.0%) |  |  |  |  |  |  |  |
|  | Deaths 4 (16%) |  |  |  |  |  |  |  |
| **Severe** | N=68 | N=27 |  |  |  |  |  |  |
| **pneumonia** | Access 51 (75%) | Access 14 (52%) |  |  |  |  |  |  |
|  | Watch 47 (69%) | Watch 27 (100%) |  |  |  |  |  |  |
|  | Reserve 0 | Reserve 0 |  |  |  |  |  |  |
|  | Deaths 17 (25%) | Deaths 10 (37%) |  |  |  |  |  |  |
| **Severe** | N=223 | N=38 | N=233 |  |  |  |  |  |
| **malnutrition** | Access 187 (84%) | Access 22 (58%) | Access 201 (86%) |  |  |  |  |  |
|  | Watch 135 (61%) | Watch 37 (97%) | Watch 134 (58%) |  |  |  |  |  |
|  | Reserve 4 (1.8%) | Reserve 2 (5.3%) | Reserve 5 (2.2%) |  |  |  |  |  |
|  | Deaths 37 (17%) | Deaths 9 (24%) | Deaths 42 (18%) |  |  |  |  |  |
| **Malaria** | N=37 | N=13 | N=52 | N=145 |  |  |  |  |
|  | Access 33 (89%) | Access 11 (85%) | Access 43 (83%) | Access 133 (92%) |  |  |  |  |
|  | Watch 18 (49%) | Watch 10 (77%) | Watch 27 (52%) | Watch 55 (38%) |  |  |  |  |
|  | Reserve 0 | Reserve 0 | Reserve 0 | Reserve 0 |  |  |  |  |
|  | Deaths 4 (11%) | Deaths 1 (7.7%) | Deaths 11 (21%) | Deaths 11 (7.6%) |  |  |  |  |
| **Moderate/** | N=244 | N=65 | N=415 | N=859 | N=369 |  |  |  |
| **severe anaemia** | Access 201 (82%) | Access 42 (65%) | Access 358 (86%) | Access 770 (90%) | Access 280 (76%) |  |  |  |
|  | Watch 130 (53%) | Watch 59 (91%) | Watch 206 (50%) | Watch 347 (40%) | Watch 138 (37%) |  |  |  |
|  | Reserve 3 (1.2%) | Reserve 1 (1.5%) | Reserve 4 (1.0%) | Reserve 6 (0.7%) | Reserve 0 |  |  |  |
|  | Deaths 36 (15%) | Deaths 14 (22%) | Deaths 54 (13%) | Deaths 106 (12%) | Deaths 22 (6.0%) |  |  |  |
| **Diarrhoea** | N=267 | N=40 | N=241 | N=889 | N=189 | N=981 |  |  |
|  | Access 207 (78%) | Access 20 (50%) | Access 208 (86%) | Access 799 (90%) | Access 155 (82%) | Access 814 (83%) |  |  |
|  | Watch 147 (55%) | Watch 40 (100%) | Watch 120 (50%) | Watch 292 (33%) | Watch 69 (37%) | Watch 346 (35%) |  |  |
|  | Reserve 3 (1.1%) | Reserve 0 | Reserve 2 (0.8%) | Reserve 5 (0.6%) | Reserve 0 | Reserve 4 (0.4%) |  |  |
|  | Deaths 30 (11%) | Deaths 4 (10%) | Deaths 35 (15%) | Deaths 95 (11%) | Deaths 16 (8.5%) | Deaths 90 (9.2%) |  |  |
| **None of these diagnoses** | N=28 | N=12 | N=102 | N=85 | N=15 | N=138 | N=295 |  |
|  | Access 23 (82%) | Access 5 (42%) | Access 85 (83%) | Access 81 (95%) | Access 8 (53%) | Access 105 (76%) | Access 168 (57%) |  |
|  | Watch 8 (29%) | Watch 11 (92%) | Watch 38 (37%) | Watch 19 (22%) | Watch 5 (33%) | Watch 59 (43%) | Watch 64 (22%) |  |
|  | Reserve 0 | Reserve 0 | Reserve 1 (1.0%) | Reserve 0 | Reserve 0 | Reserve 0 | Reserve 0 |  |
|  | Deaths 0 | Deaths 0 | Deaths 3 (2.9%) | Deaths 1 (1.2%) | Deaths 0 | Deaths 0 | Deaths 2 (0.7%) |  |

## Supplementary Table S3: Distribution of antibiotics prescribed for children admitted stratified by site at any time during their admission.

| **Characteristic** | **Banfora**, N = 431 | **Blantyre**, N = 333 | **Kampala**, N = 476 | **Kilifi** N=245 | **Migori**, N =280 | **Nairobi**, N = 279 | **Karachi**, N = 349 | **Dhaka**, N=394 | **Matlab**, N = 314 | **Overall**  N =3,101 |  |
| --- | --- | --- | --- | --- | --- | --- | --- | --- | --- | --- | --- |
| **Prior to hospitalization** | | | | | | | | | | | |
| Prior antibiotics taken, yes | 210 (49%) | 142 (43%) | 223 (47%) | 95 (39%) | 91 (33%) | 120 (43%) | 135 (39%) | 227 (58%) | 179 (57%) | 1,422 (46%) |  |
| **AWARE categories** | | | | | | | | | | | |
| Access | 375 (87%) | 242 (73%) | 410 (86%) | 198 (81%) | 237 (85%) | 262 (94%) | 210 (60%) | 365 (93%) | 178 (57%) | 2,477 (80%) |  |
| Watch | 174 (40%) | 77 (23%) | 170 (36%) | 79 (32%) | 71 (25%) | 143 (51%) | 264 (76%) | 72 (18%) | 42 (13%) | 1,092 (35%) |  |
| Reserve | 0 (0%) | 0 (0%) | 0 (0%) | 0 (0%) | 0 (0%) | 0 (0%) | 12 (3.4%) | 0 (0%) | 0 (0%) | 12 (0.4%) |  |
| **Antibiotic line** | | | | | | | | | | | |
| First line | 374 (87%) | 241 (72%) | 410 (86%) | 196 (80%) | 236 (84%) | 263 (94%) | 46 (13%) | 363 (92%) | 178 (57%) | 2,307 (74%) |  |
| Second line | 177 (41%) | 78 (23%) | 180 (38%) | 85 (35%) | 75 (27%) | 139 (50%) | 344 (99%) | 70 (18%) | 43 (14%) | 1,191 (38%) |  |
| Third line | 0 (0%) | 0 (0%) | 0 (0%) | 1 (0.4%) | 0 (0%) | 2 (0.7%) | 22 (6.3%) | 30 (7.6%) | 7 (2.2%) | 62 (2.0%) |  |
| **Antibiotic line, IV** | | | | | | | | | | | |
| First line, IV | 356 (83%) | 201 (60%) | 408 (86%) | 191 (78%) | 225 (80%) | 256 (92%) | 42 (12%) | 279 (71%) | 153 (49%) | 2,111 (68%) |  |
| second_iv | 153 (35%) | 75 (23%) | 165 (35%) | 77 (31%) | 70 (25%) | 135 (48%) | 344 (99%) | 45 (11%) | 21 (6.7%) | 1,085 (35%) |  |
| third_iv | 0 (0%) | 0 (0%) | 0 (0%) | 1 (0.4%) | 0 (0%) | 2 (0.7%) | 13 (3.7%) | 28 (7.1%) | 7 (2.2%) | 51 (1.6%) |  |
| **Antibiotic line, oral** | | | | | | | | | | | |
| First line, oral | 216 (50%) | 124 (37%) | 27 (5.7%) | 43 (18%) | 68 (24%) | 66 (24%) | 5 (1.4%) | 227 (58%) | 38 (12%) | 814 (26%) |  |
| second_oral | 49 (11%) | 9 (2.7%) | 27 (5.7%) | 25 (10%) | 13 (4.6%) | 4 (1.4%) | 6 (1.7%) | 28 (7.1%) | 23 (7.3%) | 184 (5.9%) |  |
| third_oral | 0 (0%) | 0 (0%) | 0 (0%) | 0 (0%) | 0 (0%) | 0 (0%) | 0 (0%) | 3 (0.8%) | 0 (0%) | 3 (<0.1%) |  |
| **Received antibiotics** | | | | | | | | | | | |
| Received antibiotics, yes | 405 (94%) | 260 (78%) | 465 (98%) | 227 (93%) | 255 (91%) | 278 (100%) | 347 (99%) | 381 (97%) | 200 (64%) | 2,818 (91%) |  |
| Received IV antibiotics, yes | 400 (93%) | 235 (71%) | 465 (98%) | 221 (90%) | 248 (89%) | 278 (100%) | 347 (99%) | 291 (74%) | 159 (51%) | 2,644 (85%) |  |
| Received oral antibiotics, yes | 246 (57%) | 128 (38%) | 48 (10%) | 61 (25%) | 78 (28%) | 70 (25%) | 16 (4.6%) | 243 (62%) | 59 (19%) | 949 (31%) |  |
| **Antibiotic types** | | | | | | | | | | | |
| Penicillins | 332 (77%) | 205 (62%) | 407 (86%) | 191 (78%) | 226 (81%) | 228 (82%) | 14 (4.0%) | 361 (92%) | 176 (56%) | 2,140 (69%) |  |
| Aminoglycosides | 184 (43%) | 199 (60%) | 403 (85%) | 135 (55%) | 202 (72%) | 250 (90%) | 50 (14%) | 279 (71%) | 150 (48%) | 1,852 (60%) |  |
| Penicillins & aminoglycosides | 122 (28%) | 189 (57%) | 401 (84%) | 134 (55%) | 198 (71%) | 211 (76%) | 0 (0%) | 276 (70%) | 149 (47%) | 1,680 (54%) |  |
| Third generation cephalosporins | 159 (37%) | 74 (22%) | 160 (34%) | 73 (30%) | 67 (24%) | 134 (48%) | 253 (72%) | 45 (11%) | 25 (8.0%) | 990 (32%) |  |
| Trimethoprim sulfonamide | 11 (2.6%) | 98 (29%) | 16 (3.4%) | 21 (8.6%) | 37 (13%) | 25 (9.0%) | 0 (0%) | 3 (0.8%) | 1 (0.3%) | 212 (6.8%) |  |
| Imidazoles | 46 (11%) | 18 (5.4%) | 9  (1.9%) | 8 (3.3%) | 35 (13%) | 11 (3.9%) | 31 (8.9%) | 11 (2.8%) | 3 (1.0%) | 172 (5.5%) |  |
| Amphenicols | 0 (0%) | 0 (0%) | 2 (0.4%) | 29 (12%) | 1 (0.4%) | 0 (0%) | 0 (0%) | 0 (0%) | 0 (0%) | 32 (1.0%) |  |
| Fluoroquinolones | 2 (0.5%) | 5 (1.5%) | 1 (0.2%) | 14 (5.7%) | 4 (1.4%) | 7 (2.5%) | 14 (4.0%) | 32 (8.1%) | 12 (3.8%) | 91 (2.9%) |  |
| Macrolides | 21 (4.9%) | 3 (0.9%) | 19 (4.0%) | 9 (3.7%) | 10 (3.6%) | 36 (13%) | 6 (1.7%) | 25 (6.3%) | 13 (4.1%) | 142 (4.6%) |  |
| Beta-lactamase inhibitor | 26 (6.0%) | 3 (0.9%) | 3 (0.6%) | 6 (2.4%) | 7 (2.5%) | 0 (0%) | 140 (40%) | 0 (0%) | 0 (0%) | 185 (6.0%) |  |
| Blactam anti-pseudomonal | 0 (0%) | 0 (0%) | 2 (0.4%) | 0 (0%) | 0 (0%) | 0 (0%) | 23 (6.6%) | 0 (0%) | 0 (0%) | 25 (0.8%) |  |
| Glycopeptides | 0 (0%) | 0 (0%) | 0 (0%) | 0 (0%) | 1 (0.4%) | 0 (0%) | 66 (19%) | 0 (0%) | 1 (0.3%) | 68 (2.2%) |  |
| Lincosamides | 1 (0.2%) | 0 (0%) | 0 (0%) | 0 (0%) | 0(0%) | 0 (0%) | 0 (0%) | 0(0%) | 0 (0%) | 1 (<0.1%) |  |
| Oxazolidinones | 0 (0%) | 0 (0%) | 0 (0%) | 0 (0%) | 0 (0%) | 0 (0%) | 12 (3.4%) | 0 (0%) | 0 (0%) | 12(0.4%) |  |
| Carbapenems | 0 (0%) | 0 (0%) | 0 (0%) | 1 (0.4%) | 0 (0%) | 2 (0.7%) | 11 (3.2%) | 1 (0.3%) | 1 (0.3%) | 16 (0.5%) |  |
| **Antibiotic groups, IV** | | | | | | | | | | | |
| Penicillins, IV | 294 (68%) | 192 (58%) | 406 (85%) | 189 (77%) | 222 (79%) | 228 (82%) | 14 (4.0%) | 278 (71%) | 151 (48%) | 1,974 (64%) |  |
| Aminoglycosides, IV | 184 (43%) | 199 (60%) | 403 (85%) | 135 (55%) | 202 (72%) | 250 (90%) | 50 (14%) | 279 (71%) | 150 (48%) | 1,852 (60%) |  |
| Third generation cephalosporins, IV | 150 (35%) | 74 (22%) | 156 (33%) | 73 (30%) | 67 (24%) | 134 (48%) | 253 (72%) | 43 (11%) | 20 (6.4%) | 970 (31%) |  |
| Imidazoles, IV | 38 (8.8%) | 2  (0.6%) | 9  (1.9%) | 3 (1.2%) | 14 (5.0%) | 4  (1.4%) | 27 (7.7%) | 9 (2.3%) | 3 (1.0%) | 109 (3.5%) |  |
| Amphenicols, IV | 0 (0%) | 0 (0%) | 2 (0.4%) | 29 (12%) | 1 (0.4%) | 0 (0%) | 0 (0%) | 0 (0%) | 0 (0%) | 32 (1.0%) |  |
| Fluoroquinolones, IV | 0 (0%) | 0 (0%) | 1 (0.2%) | 0 (0%) | 2 (0.7%) | 2 (0.7%) | 14 (4.0%) | 28 (7.1%) | 7 (2.2%) | 54 (1.7%) |  |
| Blactam anti-pseudomonal, IV | 0 (0%) | 0 (0%) | 2 (0.4%) | 0 (0%) | 0(0%) | 0 (0%) | 23 (6.6%) | 0 (0%) | 0 (0%) | 25 (0.8%) |  |
| Glycopeptides, IV | 0 (0%) | 0 (0%) | 0 (0%) | 0 (0%) | 1 (0.4%) | 0 (0%) | 66 (19%) | 0 (0%) | 1 (0.3%) | 68 (2.2%) |  |
| Oxazolidinones, IV | 0 (0%) | 0 (0%) | 0 (0%) | 0 (0%) | 0 (0%) | 0 (0%) | 12 (3.4%) | 0 (0%) | 0 (0%) | 12 (0.4%) |  |
| Carbapenems, IV | 0 (0%) | 0 (0%) | 0 (0%) | 1 (0.4%) | 0 (0%) | 2 (0.7%) | 11 (3.2%) | 1 (0.3%) | 1 (0.3%) | 16 (0.5%) |  |
| **Antibiotic groups, oral** | | | | | | | | | | | |
| Penicillins, oral | 197 (46%) | 22 (6.6%) | 11 (2.3%) | 17 (6.9%) | 11 (3.9%) | 4 (1.4%) | 1 (0.3%) | 222 (56%) | 37 (12%) | 522 (17%) |  |
| Third generation cephalosporins, oral | 23 (5.3%) | 0 (0%) | 6 (1.3%) | 0 (0%) | 0(0%) | 0 (0%) | 0 (0%) | 3 (0.8%) | 6 (1.9%) | 38 (1.2%) |  |
| Trimethoprim sulfonamide, oral | 11 (2.6%) | 98 (29%) | 16 (3.4%) | 21 (8.6%) | 37 (13%) | 25 (9.0%) | 0(0%) | 3 (0.8%) | 1 (0.3%) | 212 (6.8%) |  |
| Imidazoles, oral | 14 (3.2%) | 17 (5.1%) | 0 (0%) | 6 (2.4%) | 25 (8.9%) | 8 (2.9%) | 4 (1.1%) | 2 (0.5%) | 0 (0%) | 76 (2.5%) |  |
| Fluoroquinolones, oral | 2 (0.5%) | 5 (1.5%) | 0 (0%) | 14 (5.7%) | 2 (0.7%) | 5 (1.8%) | 0 (0%) | 5 (1.3%) | 5 (1.6%) | 38 (1.2%) |  |
| Macrolides, oral | 21 (4.9%) | 3 (0.9%) | 19 (4.0%) | 9 (3.7%) | 10 (3.6%) | 36 (13%) | 6 (1.7%) | 25 (6.3%) | 13 (4.1%) | 142 (4.6%) |  |
| Beta-lactamase inhibitor, oral | 25 (5.8%) | 1 (0.3%) | 3 (0.6%) | 4 (1.6%) | 4 (1.4%) | 0(0%) | 0 (0%) | 0 (0%) | 0(0%) | 37 (1.2%) |  |
| Lincosamides, oral | 1(0.2%) | 0 (0%) | 0 (0%) | 0 (0%) | 0 (0%) | 0 (0%) | 0 (0%) | 0 (0%) | 0 (0%) | 1(<0.1%) |  |
| Oxazolidinones, oral | 0 (0%) | 0 (0%) | 0 (0%) | 0 (0%) | 0 (0%) | 0 (0%) | 0 (0%) | 0 (0%) | 0 (0%) | 0 (0%) |  |
| Frequency (%) | | | | | | | | | | | |

## Supplementary Table S4: Antimicrobials classes prescribed within 48 hours of admission by cohort nutritional status strata.

| **Characteristic** | **NW**  N = 1,120 | | | **MW**  N = 763 | **SWK**  N = 1,218 | | **Overall**  N = 3,101 | | | **Weighted proportion*** |
| --- | --- | --- | --- | --- | --- | --- | --- | --- | --- | --- |
| **AWARE categories*** | | | | | | | | | |  |
| Access | 748 (67%) | | | 585 (77%) | 1,064 (87%) | | 2,397 (77%) | | | 74% |
| Watch | 289 (26%) | | | 168 (22%) | 248 (20%) | | 705 (23%) | | | 24% |
| Reserve | 0 (0%) | | | 0 (0%) | 1 (<0.1%) | | 1 (<0.1%) | | | 0.0% |
| **First-, second- and third-line regimens given within 48 hours for sepsis^†^** | | | | | | | | | |  |
| First-line | 671 (60%) | | | 541 (71%) | 1,014 (83%) | | 2,226 (72%) | | | 68% |
| Second-line | 354 (32%) | | | 206 (27%) | 269 (22%) | | 829 (27%) | | | 29% |
| Third-line | 2 (0.2%) | | | 3 (0.4%) | 9 (0.7%) | | 14 (0.5%) | | | 0.4% |
| **Antibiotic types*** | | | | | | | | | |  |
| Penicillins | 605 (54%) | | | 497 (65%) | 946 (78%) | | 2,048 (66%) | | | 62% |
| Aminoglycosides | 440 (39%) | | | 455 (60%) | 889 (73%) | | 1,784 (58%) | | | 51% |
| Penicillins & aminoglycosides | 419 (55%) | | | 386 (34%) | 815 (67%) | | 1,620 (52%) | | | 53% |
| Co-amoxiclav | | 78 (7.0%) | 39 (5.1%) | | | 24 (2.0%) | | 141 (4.5%) | 5.4% | |
| Imidazoles | 29 (2.6%) | | | 23 (3.0%) | 58 (4.8%) | | 110 (3.5%) | | | 3.2% |
| Amphenicols | 25 (2.2%) | | | 4 (0.5%) | 0 (0%) | | 29 (0.9%) | | | 1.4% |
| Anti-pseudomonal beta-lactams ‡ | 0 (0%) | | | 1 (0.1%) | 2 (0.2%) | | 3 (<0.1%) | | | 0.1% |
| Glycopeptides | 12 (1.1%) | | | 10 (1.3%) | 23 (1.9%) | | 45 (1.5%) | | | 1.3% |
| Lincosamides | 0 (0%) | | | 0 (0%) | 0 (0%) | | 0 (0%) | | | 0.0% |
| Third generation cephalosporins | 261 (23%) | | | 152 (20%) | 237 (19%) | | 650 (21%) | | | 22% |
| Fluoroquinolone | 13 (1.2%) | | | 9 (1.2%) | 18 (1.5%) | | 40 (1.3%) | | | 1.2% |
| Macrolides | 17 (1.5%) | | | 10 (1.3%) | 3 (0.2%) | | 30 (1.0%) | | | 1.2% |
| Oxazolidinones | 0 (0%) | | | 0 (0%) | 1 (<0.1%) | | 1 (<0.1%) | | | <0.1% |
| Polymyxins | 0 (0%) | | | 0 (0%) | 0 (0%) | | 0 (0%) | | | 0.0% |
| Carbapenems | 1 (<0.1%) | | | 0 (0%) | 0 (0%) | | 1 (<0.1%) | | | 0.1% |
| Trimethoprim/sulfamethoxazole  (Prophylaxis in HIV) | 32 (2.9%) | | | 30 (3.9%) | 97 (8.0%) | | 159 (5.1%) | | | 4.3% |
| Results presented as frequency (%), or days as median (IQR). Proportions for AWARE categories and antimicrobial classes total more than 100% because 2831/3101 (91%) of children receive more than one type of antibiotic. * weighted proportions; † First-, second- and third-line agents for sepsis are defined as per WHO guidelines as defined in **Supplementary Table S1.** WHO also recommends these agents for children with severe pneumonia and severe malnutrition. Here they are applied to data from all children. Sepsis regimens is also used for severe pneumonia and severe malnutrition. ‡ Beta-lactam anti-pseudomonal antimicrobials include ampicillin/clavulanic acid or sulbactam and piperacillin/tazobactam (**Supplementary Appendix 4)** . | | | | | | | | | | |

## Supplementary Table S5: Antimicrobials by cohort nutritional status strata

| **Category of antibiotic** | **Number of children receiving at least one day of antimicrobials** | **Child-days received antimicrobials** | **Child days of admission** | **Days of antimicrobials received per 100 child-days  (95% CI)** |
| --- | --- | --- | --- | --- |
| **First-line** |  |  |  |  |
| NW | 700 (63) | 2701 | 5345 | 51 (49‒53) |
| MW | 569 (75) | 2697 | 4634 | 58 (56‒60) |
| SWK | 1038 (85) | 7236 | 11828 | 61(60‒64) |
| **Second-line** |  |  |  |  |
| NW | 429 (38) | 1899 | 5345 | 36(34‒37) |
| MW | 293 (38) | 1518 | 4634 | 33 (31‒34) |
| SWK | 469 (39) | 2866 | 11828 | 24 (23‒25) |
| **Third-line** |  |  |  |  |
| NW | 13 (1.2) | 80 | 5345 | 1.5 (1.2‒1.9) |
| MW | 9 (1.2) | 60 | 4634 | 1.3 (1.0‒1.7) |
| SWK | 40 (3.3) | 252 | 11828 | 2.1 (1.9‒2.4) |
| **Access** |  |  |  |  |
| NW | 778 (69) | 3004 | 5345 | 56(54‒58) |
| MW | 611 (80) | 2923 | 4634 | 63(61‒65) |
| SWK | 1088 (89) | 7514 | 11828 | 64 (62‒65) |
| **Watch** |  |  |  |  |
| NW | 373 (33) | 1686 | 5345 | 32 (30‒33) |
| MW | 267 (35) | 1357 | 4634 | 29 (28‒31) |
| SWK | 452 (37) | 2853 | 11828 | 24 (23‒25) |
| **Reserve** |  |  |  |  |
| NW | 1 (0.09) | 3 | 5345 | 0.0 (0.0‒0.1) |
| MW | 1 (0.13) | 7 | 4634 | 0.2 (0.1‒0.3) |
| SWK | 10 (0.82) | 53 | 11828 | 0.5 (0.3‒0.6) |

NW: not wasted N=1120; MW: moderately wasted N=763; SWK: severely wasted or kwashiorkor N=1218

## Supplementary Table S6: Antibiotic prescriptions for 1886 children with antibiotic syndromes (sepsis, severe pneumonia, and severe malnutrition)

|  | **Entire admission** | | | | **Within 48 hours of admission** | | | |
| --- | --- | --- | --- | --- | --- | --- | --- | --- |
| **Characteristic** | **NW**  N = 408 | **MW**  N = 260 | **SWK**  N = 1,218 | **Overall**  N = 1,886 | **NW**  N = 408 | **MW**  N = 260 | **SWK**  N = 1,218 | **Overall**  N = 1,886 |
| **Prior to hospitalization** | | | | |  | | | |
| Prior antibiotics taken, yes | 194(48%) | 134(52%) | 526 (43%) | 854 (45%) | 194(48%) | 134(52%) | 526 (43%) | 854 (45%) |
| **AWARE categories** | | | | |  | | | |
| Access | 327(80%) | 231(89%) | 1,088(89%) | 1,646(87%) | 317(78%) | 225(87%) | 1,064(87%) | 1,606(85%) |
| Watch | 169(41%) | 114(44%) | 452 (37%) | 735 (39%) | 119(29%) | 72 (28%) | 248 (20%) | 439 (23%) |
| Reserve | 1 (0.2%) | 1 (0.4%) | 10 (0.8%) | 12 (0.6%) | 0 (0%) | 0 (0%) | 1 (<0.1%) | 1 (<0.1%) |
| **Antibiotic line** | | | | |  | | | |
| First-line | 270(66%) | 201(77%) | 1,038(85%) | 1,509(80%) | 261(64%) | 193(74%) | 1,014(83%) | 1,468(78%) |
| Second-line | 202(50%) | 135(52%) | 469 (39%) | 806 (43%) | 165(40%) | 99 (38%) | 269 (22%) | 533 (28%) |
| Third-line | 8 (2.0%) | 5 (1.9%) | 40 (3.3%) | 53 (2.8%) | 1 (0.2%) | 2 (0.8%) | 9 (0.7%) | 12 (0.6%) |
| **Antibiotic line, IV** | | | | |  | | | |
| First-line, IV | 251(62%) | 193(74%) | 1,011(83%) | 1,455(77%) | 245(60%) | 188(72%) | 987 (81%) | 1,420(75%) |
| Second-line, IV | 197(48%) | 131(50%) | 427 (35%) | 755 (40%) | 163(40%) | 96 (37%) | 258 (21%) | 517 (27%) |
| Third-line, IV | 7 (1.7%) | 4 (1.5%) | 32 (2.6%) | 43 (2.3%) | 1 (0.2%) | 2 (0.8%) | 8 (0.7%) | 11 (0.6%) |
| **Antibiotic line, oral** | | | | |  | | | |
| First-line oral | 58 (14%) | 49 (19%) | 422 (35%) | 529 (28%) | 30 (7.4%) | 21 (8.1%) | 146 (12%) | 197 (10%) |
| Second-line oral | 20 (4.9%) | 8 (3.1%) | 80 (6.6%) | 108 (5.7%) | 4 (1.0%) | 4 (1.5%) | 12 (1.0%) | 20 (1.1%) |
| Third-line oral | 1 (0.2%) | 0 (0%) | 1 (<0.1%) | 2 (0.1%) | 0 (0%) | 0 (0%) | 0 (0%) | 0 (0%) |
| **Received antibiotics** | | | | |  | | | |
| Received antibiotics, yes | 393(96%) | 258(99%) | 1,188(98%) | 1,839(98%) | 391(96%) | 257(99%) | 1,185(97%) | 1,833(97%) |
| Received IV antibiotics, yes | 382(94%) | 254(98%) | 1,170(96%) | 1,806(96%) | 380(93%) | 254(98%) | 1,165(96%) | 1,799(95%) |
| Received oral antibiotics, yes | 76 (19%) | 57 (22%) | 473 (39%) | 606 (32%) | 34 (8.3%) | 25 (9.6%) | 157 (13%) | 216 (11%) |
| **Antibiotic types** | | | | |  | | | |
| Penicillins | 242(59%) | 184(71%) | 979 (80%) | 1,405(74%) | 235(58%) | 177(68%) | 946 (78%) | 1,358(72%) |
| Aminoglycosides | 228(56%) | 184(71%) | 915 (75%) | 1,327(70%) | 220(54%) | 176(68%) | 889 (73%) | 1,285(68%) |
| Imidazoles | 15 (3.7%) | 11 (4.2%) | 101 (8.3%) | 127 (6.7%) | 11 (2.7%) | 9 (3.5%) | 58 (4.8%) | 78 (4.1%) |
| Amphenicols | 3 (0.7%) | 2 (0.8%) | 1 (<0.1%) | 6 (0.3%) | 2 (0.5%) | 2 (0.8%) | 0 (0%) | 4 (0.2%) |
| Beta-lactamase inhibitor | 62 (15%) | 32 (12%) | 49 (4.0%) | 143 (7.6%) | 57 (14%) | 30 (12%) | 24 (2.0%) | 111 (5.9%) |
| Anti-pseudomonal beta-lactams | 3 (0.7%) | 1 (0.4%) | 17 (1.4%) | 21 (1.1%) | 0 (0%) | 1 (0.4%) | 2 (0.2%) | 3 (0.2%) |
| Lincosamides | 0 (0%) | 0 (0%) | 1 (<0.1%) | 1 (<0.1%) | 0 (0%) | 0 (0%) | 0 (0%) | 0 (0%) |
| 3^rd^Gen cephalosporins | 160(39%) | 110(42%) | 413 (34%) | 683 (36%) | 115(28%) | 69 (27%) | 237 (19%) | 421 (22%) |
| Fluoroquinolones | 9 (2.2%) | 4 (1.5%) | 54 (4.4%) | 67 (3.6%) | 4 (1.0%) | 2 (0.8%) | 18 (1.5%) | 24 (1.3%) |
| Glycopeptides | 11 (2.7%) | 9 (3.5%) | 34 (2.8%) | 54 (2.9%) | 5 (1.2%) | 7 (2.7%) | 23 (1.9%) | 35 (1.9%) |
| Macrolides | 19 (4.7%) | 16 (6.2%) | 66 (5.4%) | 101 (5.4%) | 3 (0.7%) | 3 (1.2%) | 3 (0.2%) | 9 (0.5%) |
| Carbapenems | 2 (0.5%) | 1 (0.4%) | 10 (0.8%) | 13 (0.7%) | 0 (0%) | 0 (0%) | 0 (0%) | 0 (0%) |
| Oxazolidinones | 1 (0.2%) | 1 (0.4%) | 10 (0.8%) | 12 (0.6%) | 0 (0%) | 0 (0%) | 1 (<0.1%) | 1 (<0.1%) |
| Trimethoprimsulfonamide | 14 (3.4%) | 20 (7.7%) | 131 (11%) | 165 (8.7%) | 12 (2.9%) | 15 (5.8%) | 97 (8.0%) | 124 (6.6%) |
| **Antibiotic types, IV** | | | | |  | | | |
| Penicillins, IV | 230(56%) | 180(69%) | 957 (79%) | 1,367(72%) | 225(55%) | 175(67%) | 933 (77%) | 1,333(71%) |
| Aminoglycosides, IV | 228(56%) | 184(71%) | 915 (75%) | 1,327(70%) | 220(54%) | 176(68%) | 889 (73%) | 1,285(68%) |
| Imidazoles, IV | 11 (2.7%) | 8 (3.1%) | 59 (4.8%) | 78 (4.1%) | 8 (2.0%) | 8 (3.1%) | 36 (3.0%) | 52 (2.8%) |
| Amphenicols, IV | 3 (0.7%) | 2 (0.8%) | 1 (<0.1%) | 6 (0.3%) | 2 (0.5%) | 2 (0.8%) | 0 (0%) | 4 (0.2%) |
| Anti-pseudomonal beta-lactams, IV | 3 (0.7%) | 1 (0.4%) | 17 (1.4%) | 21 (1.1%) | 0 (0%) | 1 (0.4%) | 2 (0.2%) | 3 (0.2%) |
| Third generation cephalosporins, IV | 160(39%) | 109(42%) | 403 (33%) | 672 (36%) | 115(28%) | 68 (26%) | 235 (19%) | 418 (22%) |
| Glycopeptides, IV | 11 (2.7%) | 9 (3.5%) | 34 (2.8%) | 54 (2.9%) | 5 (1.2%) | 7 (2.7%) | 23 (1.9%) | 35 (1.9%) |
| Carbapenems, IV | 2 (0.5%) | 1 (0.4%) | 10 (0.8%) | 13 (0.7%) | 0 (0%) | 0 (0%) | 0 (0%) | 0 (0%) |
| Oxazolidinones, IV | 1 (0.2%) | 1 (0.4%) | 10 (0.8%) | 12 (0.6%) | 0 (0%) | 0 (0%) | 1 (<0.1%) | 1 (<0.1%) |
| **Antibiotic types, oral** | | | | |  | | | |
| Penicillins, oral | 32 (7.8%) | 17 (6.5%) | 250 (21%) | 299 (16%) | 14 (3.4%) | 5 (1.9%) | 30 (2.5%) | 49 (2.6%) |
| Trimethoprim sulfonamide, oral | 14 (3.4%) | 20 (7.7%) | 131 (11%) | 165 (8.7%) | 12 (2.9%) | 15 (5.8%) | 97 (8.0%) | 124 (6.6%) |
| Imidazoles, oral | 4 (1.0%) | 3 (1.2%) | 49 (4.0%) | 56 (3.0%) | 3 (0.7%) | 1 (0.4%) | 24 (2.0%) | 28 (1.5%) |
| Beta-lactamase inhibitor, oral | 4 (1.0%) | 2 (0.8%) | 19 (1.6%) | 25 (1.3%) | 0 (0%) | 0 (0%) | 0 (0%) | 0 (0%) |
| Lincosamides, oral | 0 (0%) | 0 (0%) | 1 (<0.1%) | 1 (<0.1%) | 0 (0%) | 0 (0%) | 0 (0%) | 0 (0%) |
| Third generation cephalosporins, oral | 4 (1.0%) | 1 (0.4%) | 16 (1.3%) | 21 (1.1%) | 0 (0%) | 1 (0.4%) | 2 (0.2%) | 3 (0.2%) |
| Fluoroquinolones, oral | 4 (1.0%) | 0 (0%) | 22 (1.8%) | 26 (1.4%) | 2 (0.5%) | 0 (0%) | 8 (0.7%) | 10 (0.5%) |
| Macrolides, oral | 19 (4.7%) | 16 (6.2%) | 66 (5.4%) | 101 (5.4%) | 3 (0.7%) | 3 (1.2%) | 3 (0.2%) | 9 (0.5%) |
| Oxazolidinones, oral | 0 (0%) | 0 (0%) | 0 (0%) | 0 (0%) | 0 (0%) | 0 (0%) | 0 (0%) | - 1. (0%) |

## Supplementary Table S7: Antimicrobials received by hospital site.

| **Category of antibiotic** | **Number of children receiving at least one day of antimicrobials** | **Child-days received antimicrobials** | **Child days of admission** | **Days of antimicrobials received per 100 child-days (95% CI)** |
| --- | --- | --- | --- | --- |
| **First-line** |  |  |  |  |
| Kilifi (n=245) | 198 (81) | 964 | 1476 | 63 (59‒68) |
| Mbagathi (n=279) | 262 (94) | 1828 | 2620 | 70 (67‒73) |
| Migori (n=280) | 237 (85) | 1180 | 1832 | 64 (61‒68) |
| Kampala (n=476) | 410 (86) | 2429 | 4847 | 50 (48‒52) |
| Blantyre (n=333) | 242 (73) | 1129 | 1920 | 58(54‒61) |
| Karachi (n=349) | 210 (60) | 862 | 2251 | 9.1 (7.9‒10) |
| Dhaka (n=394) | 365 (93) | 2062 | 2759 | 72 (69‒76) |
| Matlab (n=314) | 178 (57) | 772 | 1357 | 57 (53‒61) |
| Banfora (n=431) | 375 (87) | 2215 | 2745 | 80 (77‒84) |
| Total First-line | 2307 (74) | 12634 | 21807 | 58 (57‒59) |
| **Second-line** |  |  |  |  |
| Kilifi (n=245) | 85 (35) | 451 | 1476 | 31 (28‒34) |
| Mbagathi (n=279) | 139 (50) | 936 | 2620 | 36 (34‒38) |
| Migori (n=280) | 75 (27) | 341 | 1832 | 19 (17‒21) |
| Kampala (n=476) | 180 (38) | 848 | 4847 | 18(16‒19) |
| Blantyre (n=333) | 78 (23) | 367 | 1920 | 19 (17‒21) |
| Karachi (n=349) | 344 (99) | 2119 | 2251 | 94(90‒98) |
| Dhaka (n=394) | 70 (18) | 394 | 2759 | 14 (13‒15) |
| Matlab (n=314) | 43 (14) | 155 | 1357 | 11(9.7‒13) |
| Banfora (n=431) | 177 (41) | 682 | 2745 | 25 (23‒27) |
| Total Second-line | 1191 (38) | 6289 | 21807 | 29 (28‒30) |
| **Third-line** |  |  |  |  |
| Kilifi (n=245) | 1 (0.4) | 13 | 1476 | 0.9 (0.4‒1.5) |
| Mbagathi (n=279) | 2 (0.7) | 13 | 2620 | 0.5 (0.3‒0.8) |
| Migori (n=280) | 0 | 0 | 0 | 0 |
| Kampala (n=476) | 0 | 0 | 0 | 0 |
| Blantyre (n=333) | 0 | 0 | 0 | 0 |
| Karachi (n=349) | 22 (6.3) | 161 | 2251 | 7.2 (6.1‒8.3) |
| Dhaka (n=394) | 30 (7.6) | 163 | 2759 | 5.9 (5.0‒6.9) |
| Matlab (n=314) | 7 (2.2) | 42 | 1357 | 3.1 (2.2‒4.2) |
| Banfora (n=431) | 0 | 0 | 0 | 0 |
| Total Third-line | 62 (2.0) | 392 | 10463 | 1.8 (1.6‒2.0) |

## Supplementary Table S8: Factors associated with receiving Second-line antimicrobials.

|  |  | **Univariate analysis** | | **Multivariable analysis** | |
| --- | --- | --- | --- | --- | --- |
|  | **Children who received second-line antibiotics (N=1191)** | **Crude Risk ratios** | **P-value** | **Adjusted Risk Ratios** | **P-value** |
| Sex, female | 522 (44) | 0.93 (0.71‒1.21) | 0.57 | ¶ |  |
| Age in months (log age) | - | 1.16 (1.03‒1.31) | 0.01 | ¶ |  |
| Enrolment strata |  |  |  |  |  |
| *NW* | 429 (36) | Reference |  | ¶ |  |
| *MW* | 293 (25) | 1.07 (0.77‒1.49) | 0.67 | ¶ |  |
| *SWK* | 469 (39) | 0.95 (0.71‒1.29) | 0.76 | ¶ |  |
| Residence |  |  |  | ¶ |  |
| *Rural* | 213 (18) | Reference |  | ¶ |  |
| *Peri-urban* | 167 (14) | 1.40 (0.99‒1.98) | 0.06 | ¶ |  |
| *Urban* | 811 (68) | 1.10 (0.54‒2.24) | 0.80 | ¶ |  |
| Tertiary hospital level | 672 (56) | 2.50 (0.21‒29.4) | 0.47 | ¶ |  |
| Prior hospital admission | 366 (31) | 1.61 (1.28‒2.04) | <0.001 | 1.19 (1.04‒1.37) | 0.01 |
| Antimicrobial <7 days prior to admission | 553 (46) | 1.33 (1.11‒1.59) | 0.002 | ¶ |  |
| Stunted (LAZ<-2) | 596 (50) | 1.01 (0.80‒1.26) | 0.96 | ¶ |  |
| HIV status |  |  |  |  |  |
| *Negative* | 1055 (89) | Reference |  | ¶ |  |
| *Exposed* | 54 (4.5) | 1.10 (0.63‒1.92) | 0.73 | ¶ |  |
| *Infected* | 45 (3.8) | 1.75 (1.19‒2.59) | 0.005 | ¶ |  |
| *Untested* | 37 (3.1) | 1.51 (0.62‒3.70) | 0.37 | ¶ |  |
| **Features at admission** |  |  |  |  |  |
| Diarrhoea | 575 (48) | 1.01 (0.66‒1.55) | 0.97 | ¶ |  |
| Sepsis^†^ | 229 (19) | 2.24 (1.64‒3.07) | <0.001 | 1.41 (1.16‒1.71) | 0.001 |
| Meningitis/encephalopathy^†^ | 97 (4.1) | 18.4 (6.59‒51.6) | <0.001 | 1.70 (1.04‒2.79) | 0.04 |
| Severe pneumonia | 373 (31) | 1.30 (0.91‒1.84) | 0.15 | 1.13 (0.97‒1.31) | 0.12 |
| Other chronic illness^\|\|^ | 146 (12) | 1.67 (0.93‒2.99) | 0.09 | 1.09 (0.93‒1.26) | 0.28 |
| WHO danger sign^§^ | 899 (75) | 1.53 (1.28‒1.82) | <0.001 | 1.22 (1.05‒1.41) | 0.008 |
| Malaria rapid diagnostic test |  |  |  |  |  |
| *Negative* | 1007 (85) | Reference |  | ¶ |  |
| *Positive* | 165 (14) | 0.92 (0.76‒1.12) | 0.43 | ¶ |  |
| *Not done* | 19 (1.6) | 1.41 (0.42‒4.80) | 0.58 | ¶ |  |
| Haemoglobin g/dL |  |  |  |  |  |
| *>110* | 203 (17) | Reference |  | Reference |  |
| *100–110* | 221 (19) | 0.88 (0.68‒1.14) | 0.32 | 0.91 (0.81‒1.02) | 0.10 |
| *70–100* | 579 (49) | 1.13 (0.93‒1.38) | 0.23 | 1.00 (0.93‒1.08) | 0.98 |
| *<70* | 188 (16) | 0.98 (0.82‒1.16) | 0.78 | 0.91 (0.81‒1.02) | 0.10 |
| Blood glucose mmol/L |  |  |  |  |  |
| *<3* | 29 (2.4) | 2.55 (1.31‒4.96) | 0.006 | 1.50 (1.09‒2.04) | 0.01 |
| *3 to 10* | 1093 (92) | Reference |  | Reference |  |
| *>10* | 69 (5.8) | 0.85 (0.53‒1.34) | 0.51 | 0.86 (0.66‒1.10) | 0.23 |
| WBC *x10^9^/L* |  |  |  |  |  |
| *<5* | 30 (2.5) | 1.65 (0.84‒3.27) | 0.15 | ¶ |  |
| *5 - 17.5* | 796 (67) | Reference |  | ¶ |  |
| *>17.5* | 365 (31) | 1.19 (0.92‒1.54) | 0.18 | ¶ |  |
| Duration of admission |  |  |  |  |  |
| *<5 days* | 493 (41) | Reference |  | Reference |  |
| *≥5 days* | 698 (59) | 2.59 (1.50‒4.49) | 0.001 | 1.50 (1.05‒2.16) | 0.03 |
| Results presented as frequency (%), or risk ratios. † clinician diagnosis; \|\| Chronic illnesses including thalassemia, cerebral palsy, sickle cell disease, congenital cardiac diseases and known tuberculosis; LAZ: length for age Z score; § presence of any WHO danger signs: obstructed breathing, respiratory distress, cyanosis, shock, severe anaemia, convulsions, severe dehydration, profuse watery diarrhoea, vomiting everything, Impaired consciousness, temperature >38°C in last 24 hrs or <36°C in last 24h; LAZ = length-for-age Z score; ¶ variables not selected for inclusion in multivariable model using backward stepwise selection; NW, no wasting; MW, moderate wasting; SWK, severe wasting or kwashiorkor (severe malnutrition). Risk ratios from multilevel mix-effects generalized linear model with site as random intercept and including the sampling weights, multivariable AUC 0.80 (95%CI 0.78‒0.82) | | | | | |

Appendix 1: Enrolment nutritional strata matrix

|  |  | **Criteria** | |
| --- | --- | --- | --- |
| **Nutritional group** | **Metric** | **≥6 months of age** | **<6 months of age** |
| **1- Not wasted (NW)** | MUAC | ≥ 125 mm | ≥ 120 mm |
| **2- Moderate wasting (MW)** | MUAC | <125 mm to ≥115 mm | <125 mm to ≥115 mm |
| **3- Severe wasting or   nutritional oedema/Kwashiorkor (SWK)** | MUAC | <115 mm | <110 mm |
|  | Evaluation of oedema | Mild | Mild |
|  |  | Moderate | Moderate |
|  |  | Severe | Severe |
| Mild (+) in feet only; Moderate (++) in feet and lower limbs; Severe (+++) generalized including upper body and face | | | |

# Appendix 2-Daily review CRF

| **Month __ __ __ Date** | | __ __ | __ __ | | __ __ | | __ __ | __ __ | | __ __ | __ __ | |
| --- | --- | --- | --- | --- | --- | --- | --- | --- | --- | --- | --- | --- |
| (24h clock) **Time seen** | | __ __ : __ __ | __ __ : __ __ | | __ __ : __ __ | | __ __ : __ __ | __ __ : __ __ | | __ __ : __ __ | __ __ : __ __ | |
| **Weight *kg* now** | | __ __ · __ __ 🞏Too sick | __ __ · __ __ 🞏Too sick | | __ __ · __ __ 🞏Too sick | | __ __ · __ __ 🞏Too sick | __ __ · __ __ 🞏Too sick | | __ __ · __ __ 🞏Too sick | __ __ · __ __ 🞏Too sick | |
| **MUAC *cm* now** | | __ __ · __ | __ __ · __ | | __ __ · __ | | __ __ · __ | __ __ · __ | | __ __ · __ | __ __ · __ | |
| **Oedema now** | | +++ ++ + N | +++ ++ + N | | +++ ++ + N | | +++ ++ + N | +++ ++ + N | | +++ ++ + N | +++ ++ + N | |
| **Oedema improving?** | | Y N | Y N | | Y N | | Y N | Y N | | Y N | Y N | |
| **Clinical Events in the last 24h** | | | | | | | | | | | | |
|  | **Obstructed breathing** | 🞏 | 🞏 | 🞏 | | 🞏 | | | 🞏 | 🞏 | | 🞏 |
| **DANGER SIGNS  at any time in last 24h?**  *If any new danger signs take bloods and record these in sample log. If bloods have been taken by clinical team document results, do not retake bloods* | **Respiratory distress** | 🞏 | 🞏 | 🞏 | | 🞏 | | | 🞏 | 🞏 | | 🞏 |
|  | **Cyanosis** | 🞏 | 🞏 | 🞏 | | 🞏 | | | 🞏 | 🞏 | | 🞏 |
|  | **Shock*** | 🞏 | 🞏 | 🞏 | | 🞏 | | | 🞏 | 🞏 | | 🞏 |
|  | **Severe anaemia*** | 🞏 | 🞏 | 🞏 | | 🞏 | | | 🞏 | 🞏 | | 🞏 |
|  | **Convulsion(s)** | 🞏 | 🞏 | 🞏 | | 🞏 | | | 🞏 | 🞏 | | 🞏 |
|  | **Severe Dehydration** | 🞏 | 🞏 | 🞏 | | 🞏 | | | 🞏 | 🞏 | | 🞏 |
|  | **Profuse watery Diarrhoea** | 🞏 | 🞏 | 🞏 | | 🞏 | | | 🞏 | 🞏 | | 🞏 |
|  | **Vomits everything** | 🞏 | 🞏 | 🞏 | | 🞏 | | | 🞏 | 🞏 | | 🞏 |
|  | **Impaired Consciousness** | 🞏 | 🞏 | 🞏 | | 🞏 | | | 🞏 | 🞏 | | 🞏 |
| **Temperature >38^O^C in last 24h** | | Y N | Y N | Y N | | Y N | | | Y N | Y N | | Y N |
| **Temperature <36^O^C in last 24h** | | Y N | Y N | Y N | | Y N | | | Y N | Y N | | Y N |
| **NG tube in last 24h** | | Y N | Y N | Y N | | Y N | | | Y N | Y N | | Y N |
| **Any EBM or breastfeeding in 24h** | | Y N | Y N | Y N | | Y N | | | Y N | Y N | | Y N |
| **ReSoMal in last 24h** | | Y N | Y N | Y N | | Y N | | | Y N | Y N | | Y N |
| **ORS in last 24h** | | Y N | Y N | Y N | | Y N | | | Y N | Y N | | Y N |
| **IV fluids given in last 24h** | | Y N | Y N | Y N | | Y N | | | Y N | Y N | | Y N |
| **Blood transfusion given in last 24h** | | Y N | Y N | Y N | | Y N | | | Y N | Y N | | Y N |
| **Oxygen given in last 24h?** | | Y N | Y N | Y N | | Y N | | | Y N | Y N | | Y N |
| **CPAP given in last 24h?** | | Y N | Y N | Y N | | Y N | | | Y N | Y N | | Y N |
| **Clinical Observations now** | | | | | | | | | | | | |
| **Oxygen saturation now** | | __ __ __% | __ __ __% | __ __ __% | | __ __ __% | | | __ __ __% | __ __ __% | | __ __ __% |
| **Respiratory rate now** | | __ __ __/min | __ __ __/min | __ __ __/min | | __ __ __/min | | | __ __ __/min | __ __ __/min | | __ __ __/min |
| **Heart rate now** | | __ __ __/min | __ __ __/min | __ __ __/min | | __ __ __/min | | | __ __ __/min | __ __ __/min | | __ __ __/min |
| **AVPU now** (circle) | | A V P U | A V P U | A V P U | | A V P U | | | A V P U | A V P U | | A V P U |
| **Temperature now** | | __ __ . __ ^o^C | __ __ . __ ^o^C | __ __ . __ ^o^C | | __ __ . __ ^o^C | | | __ __ . __ ^o^C | __ __ . __ ^o^C | | __ __ . __ ^o^C |
| **In PICU/ HDU now** | | Y N | Y N | Y N | | Y N | | | Y N | Y N | | Y N |
| **In a surgical or specialist unit now** | | Y N | Y N | Y N | | Y N | | | Y N | Y N | | Y N |
| **Currently has an IV cannula?** | | Y N | Y N | Y N | | Y N | | | Y N | Y N | | Y N |
| **Currently IV antibiotic 1^st^/2^nd^/3^rd^ line?** | | 1 2 3 N | 1 2 3 N | 1 2 3 N | | 1 2 3 N | | | 1 2 3 N | 1 2 3 N | | 1 2 3 N |
| **Currently anti-TB treatment?** | | Y N | Y N | Y N | | Y N | | | Y N | Y N | | Y N |
| **Currently on F75/equivalent** | | Y N | Y N | Y N | | Y N | | | Y N | Y N | | Y N |
| **Currently on F100/equivalent** | | Y N | Y N | Y N | | Y N | | | Y N | Y N | | Y N |
| **Currently on RUTF** | | Y N | Y N | Y N | | Y N | | | Y N | Y N | | Y N |
| **Currently on supplementary feed** | | Y N | Y N | Y N | | Y N | | | Y N | Y N | | Y N |
| **Infant formula or dilute F100** | | Y N | Y N | Y N | | Y N | | | Y N | Y N | | Y N |
| **Non-standard milk e.g. soya** | | Y N | Y N | Y N | | Y N | | | Y N | Y N | | Y N |
| **Clinician initials** | |  |  |  | |  | | |  |  | |  |

Clinical deterioration = developing any new danger signs, new hypothermia or pyrexia, new antibiotics started/changed, given blood transfusion, new oxygen requirement, admission to ICU/HDU. Severe anaemia = haemoglobin <4g/dL or respiratory signs and haemoglobin 4-6g/dL

Shock = cool peripheries, fast week pulse and capillary refill time >2s

| **INITIAL TREATMENT** | | | | | | | |
| --- | --- | --- | --- | --- | --- | --- | --- |
| Admitted to: *select one* | 🞏 Admission to ward | | 🞏 Admission to HDU | | | 🞏 Admission to ICU | |
| **Date and time First antibiotics given** | __ __ / __ __ / __ __ __ __ ___ ___:___ ___ *24h clock* | | | | | | 🞏Not given |
| **Intravenous Antibiotics Given?**  **🞏 Not given** | 🞏 Penicillin | 🞏 Gentamicin | | | 🞏 Ceftriaxone | | |
|  | 🞏 Co-amoxiclav | 🞏 Flu/Cloxacillin | | | 🞏 Chloramphenicol | | |
|  | 🞏 Ampicillin | 🞏 Amikacin | | | 🞏 Meropenem | | |
|  | 🞏 Levofloxacin | 🞏 Vancomycin | | | 🞏 Metronidazole | | |
|  | 🞏 Other_________________________________________________________ | | | | | | |
| **Oral Antibiotics Given?**  **🞏 Not given** | 🞏 Amoxicillin | 🞏 Erythromycin | | | 🞏 Azithromycin | | |
|  | 🞏 Co-trimoxazole | 🞏 Metronidazole | | | 🞏 Ciprofloxacin | | |
|  | 🞏 Cefalexin / cefaclor | 🞏 Co-amoxiclav | | | 🞏 Nalidixic acid | | |
|  | 🞏Penicillin | 🞏 Flucloxacillin | | | 🞏 Levofloxacin | | |
|  |  |  | | | 🞏 Other ______________ | | |
| **Initial treatment given.**  *First 6 hours.  Select any that apply.*  *For IV fluid bolus, and IV fluids specify type and volume in ml, and duration* | 🞏 IV Fluid Bolus | | | 🞏 IV Maintenance Fluids | | | |
|  | 🞏 Oxygen | | | 🞏 Warmth (heater, warmed fluids) | | | |
|  | 🞏 IV Glucose 🞏 Oral Glucose | | | 🞏 Commercial F75 | | | |
|  | 🞏 Blood transfusion | | | 🞏 Commercial F100 | | | |
|  | 🞏 Phenobarbitone | | | 🞏 Locally prepared F75/ milk suji | | | |
|  | 🞏 Diazepam | | | 🞏 Local prepared F100 / milk suji 100 | | | |
|  | 🞏 Paracetamol | | | 🞏 Expressed breast milk | | | |
|  | 🞏 Ibuprofen | | | 🞏 Dilute F100 | | | |
|  | 🞏 Antimalarial | | | 🞏 Other milk/ formula/ feed | | | |
|  | 🞏 ReSoMal | | | 🞏 Other | | | |
|  | 🞏 ORS | | | _______________________________ | | | |

# Appendix 3-Initial treatment CRF

# Antimicrobial classification matrix

| **Admission antibiotics** | | | | |
| --- | --- | --- | --- | --- |
| **Oral antibiotic** | **Antibiotic** | **Group** | **Line** | **AWARE classification** |
|  | Amoxicillin | Penicillins | 1st Line | Access |
|  | Penicillin | Penicillins | 1st Line | Access |
|  | Flucloxacillin | Penicillins | 1st Line | Access |
|  | Pivmecillinam | Penicillins | 1st Line | Access |
|  | Metronidazole | imidazole | 1st Line | Access |
|  | Cotrimoxazole | Trimethoprim - sulphonamide combinations | 1st Line | Access |
|  | Erythromycin | Macrolide | 1st Line | Watch |
|  | Nalidixic acid | Quinolones/Fluoroquinolones | 1st Line | Watch |
|  | Cefalexin | First-generation cephalosporins | 2nd Line | Access |
|  | Co-amoxiclav | Beta lactam - beta lactamase inhibitor | 2nd Line | Access |
|  | Azithromycin | Macrolide | 2nd Line | Watch |
|  | Ciprofloxacin | Quinolones/Fluoroquinolones | 2nd Line | Watch |
|  | Cefaclor | Second-generation cephalosporins | 2nd Line | Watch |
|  | Levofloxacin | Quinolones/Fluoroquinolones | 3rd Line | Watch |
| **IV antibiotics** | | | | |
|  | Benzylpenicillin | Penicillins | 1st Line | Access |
|  | Ampicillin | Penicillins | 1st Line | Access |
|  | Flucloxacillin | Penicillins | 1st Line | Access |
|  | Gentamicin | [Aminoglycosides](https://en.wikipedia.org/wiki/Aminoglycoside) | 1st Line | Access |
|  | Chloramphenicol | Amphenicol | 1st line | Access |
|  | Metronidazole | imidazole | 1st Line | Access |
|  | Amikacin | Aminoglycosides | 2nd Line | Access |
|  | Co-amoxiclav / Pipericillin Tazobactam/ Sulbactam | Beta lactam - beta lactamase inhibitor | 2nd Line | Access |
|  | Vancomycin | Glycopeptides | 2nd Line | Watch |
|  | Ceftriaxone/Cefotaxime/Ceftazidime | Third generation cephalosporins | 2nd Line | Watch |
|  | Ceftazidime | Third generation cephalosporins | 2nd Line | Watch |
|  | Meropenem/Imipenem | Carbapenems | 3rd Line | Watch |
|  | Levofloxacin | Quinolones/Fluoroquinolones | 3rd Line | Watch |
|  | Linezolid | Oxazolidinones | 3rd Line | Reserve |

# Appendix 4: WHO treatment guidelines for sepsis and Meningitis as per 2013 WHO Pocketbook of hospital care for children: guidelines for the management of common childhood illnesses.

**Serious Bacterial Infection**

New-borns with documented risk factors are more likely to develop serious bacterial infection. Some danger signs of serious bacterial listed below:

1. Danger signs: Not feeding well, convulsions, drowsy or unconscious, movement only when stimulated or no movement at all, fast breathing (60 breaths per min), grunting, severe chest indrawing, raised temperature > 38°C, hypothermia, < 35.5°C and central cyanosis.
2. Severe jaundice
3. Severe abdominal distension

Localizing signs of infection are:

1. Signs of pneumonia (Fever or hypothermia, Shock (lethargy, fast breathing, cold skin, prolonged capillary refill, fast weak pulse, and sometimes low blood pressure) and seriously ill with no apparent cause
2. Many or severe skin pustules
3. Umbilical redness extending to the peri-umbilical skin.
4. Umbilicus draining pus.
5. Bulging fontanelle
6. Painful joints, joint swelling, reduced movement, and irritability if these parts are handled.

**Treatment**

*Antibiotic therapy*

Empirical antibiotics should be given to children with suspected neonatal sepsis.

- Admit to hospital.
- When possible, do a lumbar puncture and obtain blood cultures before starting antibiotics.
- For new-borns with any signs of serious bacterial infection or sepsis, give ampicillin (or penicillin) and gentamicin as first-line antibiotic treatment.
- If at greater risk of staphylococcus infection (extensive skin pustules, abscess, or omphalitis in addition to signs of sepsis), give IV cloxacillin and gentamicin.
- The most serious bacterial infections in newborns should be treated with antibiotics for at least 7–10 days.
- If an infant is not improving within 2–3 days, change the antibiotic treatment or refer the infant for further management.

**Meningitis**

Clinical signs

Suspect meningitis if signs of serious bacterial infection are present, particularly if any one of the following is present:

The infant is:

- Drowsy, lethargic, or unconscious
- Convulsing
- Has a bulging fontanelle
- Irritable
- Has a high-pitched cry.

It is important to attempt lumbar puncture once the infant has been stabilized,

ideally within 2 h of initiating antibiotic treatment, because it serves to confirm the diagnosis.

*Treatment*

- The first-line antibiotics are ampicillin and gentamicin for 3 weeks.
- Alternatively, give a third-generation cephalosporin, such as ceftriaxone (50 mg/kg every 12 h if < 7 days of age and 75 mg/kg after 1 week) or
- Cefotaxime (50 mg/kg every 12 h if < 7 days or every 6–8 h if > 7 days of age), and gentamicin for 3 weeks.
- If there are signs of hypoxaemia, give oxygen.
- If the infant is drowsy or unconscious, ensure that hypoglycaemia is not present; if it is, give 2 ml/kg 10% glucose IV.
- Treat convulsions (after ensuring they are not due to hypoglycaemia or hypoxemia) with phenobarbital.
- Make regular checks for hypoglycaemia.

**Choices of antibiotic recommendations for various diagnoses**

| **Choices of antibiotic recommendations for various diagnoses** |  | **Dosage** |
| --- | --- | --- |
| **Sepsis in a child aged <2 months** |  |  |
| Ampicillin intravenous plus gentamicin intravenous | 50 mg/kg QID for 7–10 days (21 days for meningitis) |  |
| Second line: ceftriaxone intravenous | 50–100 mg/kg once daily for 7–10 days |  |
| **Sepsis in a child aged <2 months where referral is not possible** |  |  |
| Amoxicillin oral plus gentamicin intramuscular or intravenous | 50 mg/kg BID for 7 days | 5–7·5 mg/kg daily for 2–7 days |
| **Sepsis in a child aged <2 months if skin conditions suggest *Staphylococcus aureus*** |  |  |
| Cloxacillin or flucloxacillin intravenous plus gentamicin | 25–50 mg/kg BID or QID (age dependent) for 7–10 days | 5–7·5 mg/kg daily for 7–10 days (21 days for meningitis) |
| **Sepsis in a child aged >2 months** |  |  |
| Ampicillin intravenous plus gentamicin intravenous or intramuscular | 50 mg/kg QID for 7–10 days | 7·5 mg/kg daily for 7–10 days |
| Second line: ceftriaxone intravenous or intramuscular | 50 mg/kg BID or 100 mg/kg daily for 7–10 days |  |
| **Sepsis in a child aged >2 months if skin conditions suggest *S aureus*** |  |  |
| Flucloxacillin intravenous plus gentamicin | 50 mg/kg QID for 7–10 days |  |
| **Typhoid fever** |  |  |
| Ciprofloxacin oral | 15 mg/kg BID for 7–10 days |  |
| Second line: intravenous ceftriaxone or azithromycin oral | 80–100 mg/kg daily for 5–7 days | 20 mg/kg daily for 5–7 days |
| **Pneumonia** |  |  |
| Ampicillin intravenous plus gentamicin intravenous | 50 mg/kg QID for 7–10 days | 7·5 mg/kg daily for 7–10 days |
| Second line: ceftriaxone intravenous | 80 mg/kg daily for 7–10 days |  |
| **Pneumonia (if *S aureus* is suspected)** |  |  |
| Flucloxacillin or cloxacillin intravenous plus gentamicin | 50 mg/kg QID for 7–10 days | 7·5 mg/kg intramuscular or intravenous once a day |
| **Dysentery (presumed due to *Shigella* spp)** |  |  |
| Ciprofloxacin oral | 15 mg/kg BID for 3 days |  |
| Second line: ceftriaxone intravenous | 50–80 mg/kg daily for 3 days |  |
| **Osteomyelitis** |  |  |
| Chloramphenicol | 25 mg/kg TID |  |
| Second line: cloxacillin or flucloxacillin intravenous or clindamycin or third generation cephalosporins | 50 mg/kg QID for up to 5 weeks (step down to oral once clinically improving) | No dosages specified; clear circumstances of when such therapy would be appropriate are not outlined |
| **Meningitis in neonates** |  |  |
| Ampicillin plus gentamicin | 50 mg/kg BID for 3 weeks | 5–7·5 mg/kg daily for 3 weeks |
| Ceftriaxone intravenous | 50–75 mg/kg daily for 3 weeks | 5–7·5 mg/kg daily for 3 weeks |
| Cefotaxime plus gentamicin | 50 mg/kg BID or TID (age dependent) for 3 weeks | 5–7·5 mg/kg daily for 3 weeks |
| **Meningitis in children older than 28 days** |  |  |
| Ceftriaxone intravenous | 50 mg/kg intramuscular or intravenous BID for 7–10 days |  |
| Second line: cefotaxime intravenous | 50 mg/kg intramuscular or intravenous QID for 7–10 days |  |
| **Meningitis in children older than 28 days with no known resistance to chloramphenicol or β-lactams locally** | | 50 mg/kg QID for 10 days |
| Chloramphenicol intravenous plus ampicillin intramuscular or intravenous or benzylpenicillin intravenous | 25 mg/kg QID for 10 days | 60 mg/kg QID for 10 days |
| **Urinary tract infection** |  |  |
| Co-trimoxazole oral | 4 mg/kg plus 20 mg/kg BID for 5 days |  |
| Second line: ampicillin plus gentamicin | 50 mg/kg intramuscular or intravenous every 6h |  |
|  |  | 5–7·5 mg/kg daily |
| First-line and second-line treatment guidelines for common paediatric infective illnesses. Data are from WHO pocket book of hospital care for children and WHO guideline for managing possible serious bacterial infection in young infants when referral is not feasible BID=twice daily. TID=three times daily. QID=four times daily. | | |

# Appendix 5: Statistical Analysis Plan

**Antibiotic prescribing patterns in acutely ill hospitalised children aged 2-23 months in Sub-Saharan Africa.**

| **Version Control** | **Update after any change** |
| --- | --- |
| **Version 1.0** | **Created by Caroline Tigoi** |
| **Version 1.1** | **Comments from Moses Ngari** |
| **Version 1.2** | **Comments from Jay Berkley** |
| **Version 1.3** | **Comments From Nicole Stoesser** |

This statistical analysis plan is prepared according to **STROBE** statement checklist for cohort studies.

Table of Contents

[1. Background 3](#_Toc83204679)

[1.1 Research questions 3](#_Toc83204680)

[2. Objectives 4](#_Toc83204681)

[2.1 General objective 4](#_Toc83204682)

[2.2 Specific objectives 4](#_Toc83204683)

[3. Study Design 5](#_Toc83204698)

[3.1 Setting 5](#_Toc83204699)

[3.2 Participants 6](#_Toc83204700)

[3.3 Stratification 6](#_Toc83204701)

[3.4 Timelines 7](#_Toc83204702)

[3.5 Variables 7](#_Toc83204703)

[3.6 Outcomes 7](#_Toc83204704)

[3.7 Exposures 7](#_Toc83204705)

[3.8 Potential confounders and effect modifiers 8](#_Toc83204706)

[3.9 Bias (adopted from CHAIN) 8](#_Toc83204707)

[3.10 Study Size 9](#_Toc83204708)

[3.11 Quantitative variables 9](#_Toc83204709)

[4. Statistical methods 10](#_Toc83204710)

[4.1 Participants 10](#_Toc83204711)

[4.2 Baseline characteristics 10](#_Toc83204712)

[4.3 Outcome data 10](#_Toc83204713)

[4.4 Main results 11](#_Toc83204714)

[4.5 Summary of analyses 11](#_Toc83204717)

[4.6 Regression analysis 12](#_Toc83204718)

[5. References 12](#_Toc83204719)

[6. Appendix 1: Domains 14](#_Toc83204720)

[7. Appendix 2: STROBE Statement 15](#_Toc83204721)

1. **Background**

Antibiotics are indispensable in modern medicine to treat infections caused by bacteria[1]. Irrational antibiotic prescribing poses a serious public health problem contributing to the development of antimicrobial resistance (AMR) which contributes to negating the remarkable benefits gained from the development of antibiotics. Antibiotics are the most prescribed drugs in hospitals[2], with up to 60% being used inappropriately [3]. For example, broad-spectrum antibiotics may be chosen over narrow-spectrum options and are often delivered intravenously when oral administration may be appropriate[4]. The World Health Organization reports a clear lack in effective surveillance of AMR with poor methodology standards, data sharing and coordination[5]. Multidrug resistant organisms (MDRO) are increasingly prevalent in low middle-income countries (LMICs) due to the wide unregulated use of antibiotics with far-reaching effects. This is particularly concerning because infections with ESBL-E and CPE are difficult to treat in LMICs due to associated with more severe infections, prolonged stay in hospital and limited alternative treatment options that are costly and unavailable in these settings[6-8]. International literature suggests that up to one third of pediatric deaths may be attributable to antimicrobial resistance (AMR) in hospital and post-discharge[9]. Importantly there are no pipelines for discovery of new antibiotics that will deliver affordable agents in the next decade[10]. Thus, there is an urgent need to address this crisis of AMR and knowledge of antibiotic prescribing patterns contribute to developing standards and guidelines for prescribing and antibiotic stewardship programs[2]. This study aims to determine the antibiotic prescribing patterns in acutely ill hospitalised children aged 2-23 months in Africa.

1. **Research questions.**
2. What classes of antibiotics are given and what proportion of children are given antibiotics at admission, after 48 hours, stopped or re-introduced within 30 days of admission?
3. What is the number of hospital-days spent on each class of antibiotics and the incidence of use of second and third-line antibiotics during index admission?
4. What factors are associated with receiving specific class or group of antibiotics i.e. 1^st^ line, 2^nd^ line, aminoglycosides, penicillin, beta lactams, macrolides, monobactams, fluoroquinolones, 2^nd^ or 3^rd^ generation cephalosporins, carbapenem etc. at any time during admission?
5. **Objectives**
6. **General objective**

To describe antibiotic prescribing patterns in acutely ill hospitalised children aged 2-23 months Burkina Faso, Kenya, Malawi and Uganda.

1. **Specific objectives**
2. To determine the proportion of children admitted who receive oral or IV antibiotics at any time during their admission.
3. To describe the antimicrobials and classes prescribed at admission and introduced after at least 48 hours.
4. To determine the number of children who restart IV antibiotics within 30 days of index admission including restarting in hospital and being readmitted.
5. To determine the number of hospital-days spent on each class of antibiotics and in each WHO AWaRe category, and the incidence of use of second and third-line antibiotics.
6. To determine the factors associated with receiving specific class or groups of antibiotics 1^st^ line, 2^nd^ line, as aminoglycosides, penicillin, beta lactams, macrolides, monobactams, fluoroquinolones, 2^nd^ or 3^rd^ generation cephalosporins etc. at any time during admission.

1. **Study Design**

This is a secondary analysis of a stratified prospective cohort study of acutely ill children enrolled into the CHAIN cohort study. The children were under observation during their index hospital admission with variable duration of stay in hospital, then followed up for a fixed duration of 180 days post-discharge, [11]. Outcomes of the types and duration of antibiotics received relative to exposures including patient factors, guidelines and facility factors will be examined.

1. **Setting**

Six African sites in the CHAIN network (Burkina Faso: Banfora Referral Hospital, Kenya: Kilifi County Hospital; Mbagathi Sub-County Hospital, Nairobi; Migori County Hospital, Malawi: Queen Elizabeth Hospital, Blantyre and Uganda: Mulago Hospital, Kampala). These sites all serve vulnerable populations and represent a range of environments, populations, access and levels of background comorbidities such as malaria and HIV.

1. **Participants**

CHAIN recruited children at admission to hospital in three strata to ensure the recruitment of children with a spectrum of nutritional status. The comprehensive nature of data collection, sampling and follow up meant limiting the rate of enrolment to address data quality and workload. Participants were identified by choosing the first admissions from a specified day each week until the weekly quota for each stratum was met. Children were treated according to current national and international clinical guidelines.

Inclusion criteria were:

1. Aged 2 to 23 month.
2. Admitted to a study hospital and planning to remain in the hospital catchment area for at least 6 months and willing to come for the specified follow up visit.
3. Informed consent

Exclusion criteria were:

1. Requiring immediate resuscitation at admission to hospital
2. Unable to tolerate oral feeds while in his/her usual state of health.
3. Underlying terminal illness that in the opinion of the treating physician is likely to lead to death within 6 months (e.g., cancer, congenital heart disease)
4. Diagnosed with a condition that in the opinion of the treating physician is likely to require surgery within 6 months
5. Diagnosed chromosomal abnormality (syndromically or genetically diagnosed abnormality)
6. Primary reason for admission is poisoning, trauma or a surgical condition
7. Previously enrolled in this study
8. Sibling currently or previously enrolled in this study
9. **Stratification**

Enrolled children were stratified into three groups at admission:

- Severe Wasting/Kwashiorkor (SWK): MUAC <11.5cm (MUAC <11.0cm under 6 months old) or kwashiorkor.
- Moderate wasting (MW): MUAC 11.5 to <12.5cm at any age or MUAC 11.0 to <12.0cm under 6 months old
- Not wasted (NW): MUAC ≥12.5cm at any age or (MUAC ≥12cm or more under 6 months old

1. **Timelines**

Recruitment began on 20^th^ November 2016 and ended on 31^st^ January 2019.

1. **Variables**
2. **Outcomes**

Antibiotics received (oral/IV, classes and AWARE category), duration and repeat treatment.

1. **Exposures**

Exposure variables were collected in a standardized Case Report Form (CRF) using study standard operation procedure (SOP)s by trained staff. These includes clinical data was collected during hospitalization for all the children including demographics, daily reviews, clinical features and diagnosis, progress and treatment received.

**Patient factors**

| 1. Age | Continuous |
| --- | --- |
| 1. Gender | M/F |
| 1. Anthropometry | NW, MW, SWK and Community |
| 1. Previously admitted to hospital | - < 1 week ago - 1 week-1 month ago - >1 month ago |
| 1. Medication in the last 7 days | Y/N (since collecting data specifically on antibiotics was observed to be unreliable) |
| 1. Initial diagnosis (Include those with percentage of >5) | Infection – sepsis, pneumonia, malaria, gastroenteritis, skin and soft tissue infections, bronchiolitis, suspected meningitis, otitis media, febrile convulsions, unknown febrile illness, enteric fever and UTI |
| 1. Chronic conditions | Pulmonary TB, HIV, Sickle Cell Disease, cerebral palsy, neurological problems/epilepsy, renal impairment, congenital cardiac disease |
| 1. Illness severity | Low, Medium or High score as per the main CHAIN cohort analysis |
| 1. Available laboratory results | Blood culture – positive/negative/ unavailable   - Unavailable results - Abnormal WBC (high/low) - Malaria RDT |

**Facility features.**

| 1. Level of facility | National referral hospital, regional hospital or district/sub-regional level hospital |
| --- | --- |
| 1. Rural and urban | R/U |
| 1. Local antibiotic guidelines | Y/N (1^st^ line – Amp-Gent or Pen-Gentamicin or Benzylpenicillin, or ceftriaxone/cefuroxime if meningitis is suspected) |

**Factors for admission antibiotics and switching to 2^nd^ line – Presence of WHO danger signs at admission or within 48 hours after admission?**

| 1. Obstructed breathing | Y/N |
| --- | --- |
| 1. Respiratory distress | Y/N |
| 1. Cyanosis | Y/N |
| 1. Shock | Y/N |
| 1. Severe Anaemia | Y/N |
| 1. Convulsions | Y/N |
| 1. Severe dehydration | Y/N |
| 1. Profuse watery Diarrhoea | Y/N |
| 1. Vomiting everything | Y/N |
| 1. Impaired consciousness | Y/N |
| 1. Temperature >38^O^C in last 24 hrs | Y/N |
| 1. Temperature >36^O^C in last 24 hrs | Y/N |

1. **Potential confounders and effect modifiers**

Since this is a stratified cohort, all proportions at admission and regression analysis will include sampling weights to reflect the ratios between the strata enrolled and the actual admissions in each stratum.

1. **Bias (adopted from CHAIN)**

Selection bias: Enrollment to the study was stratified into three strata based on MUAC. This will be accounted for by weighting the analysis with the sampling weights.

Attrition and outcome assessment bias: Withdrawals or those absconding from hospital were addressed during consenting to help reduce lost to follow-up.

To reduce measurement bias, we standardised clinical care across the sites, training and assessment of clinical signs and definitions. Identical anthropometry equipment was centrally purchased, scales were calibrated, and measurements were performed by two independent observers and their arithmetic mean used in analysis. Data with implausible (absolute or relative to either other cohort participants or to the same participant measured at difference time points) were referred back to sites for resolution or set to ‘missing’ where unresolvable. A summary of implausible results will be tabulated by strata, site and age.

Missing data will be handled as follows for different type of variables:

Clinical and routine laboratory test: A `missing/test not done’ category will be added as such as for routine tests like infant HIV, malaria RDT.[14] We will not assume data were missing at random.

Socioeconomic variables: Children who died early (first 48 hours) may not have socioeconomic data collected because collection of these data was not prioritised at the time of hospitalisation. Therefore, missing these data was associated with early death. Thus, we will create a model with ‘a missing’ category for these data. A sensitivity analysis will also be conducted for complete cases only.

Laboratory variables: culture and antibiotic sensitivity testing was done using standardised SOPs in Kenyan sites as per CLSI guidelines. For other African sites available results will be coded as gram positive/negative or the AST results and if not available, then coded as not done in the analysis.

1. **Study Size**

The CHAIN cohort recruited 3,101 children (2,044 from African sites) with power of 80% to detect differences in proportion of children who would die post-discharge between non-wasted and moderately wasted children, a two-tailed alpha of 0.05 and allowing for 10% lost to follow-up. The proportion of children receiving cephalosporins in Kenyan hospitals was 26%[12], hence the overall CHAIN participants from Africa sites (N=2,044) will be adequate to explore factors associated antibiotics use with >90% power and two-tailed alpha of 0.05.

| **Proportion of children receiving cephalosporins** | **95% CI** | **Sample size** |
| --- | --- | --- |
| 25% | 19 to 32% | 237 to 335 |
| 33% | 27 to 40% | 303 to 369 |
| 50% | 43 to 57% | 377 to 377 |
| 67% | 60 to 73% | 303 to 369 |
| 75% | 68 to 80% | 246 to 335 |

1. **Quantitative variables**

Comorbid/chronic conditions will be analysed i.e. TB, HIV, sickle cell among others as binary variables.

WHO AWaRe antibiotic classification (Access, Watch or Reserve) and WHO prescribing indicators which include the average number of drugs prescribed per encounter, the percentage of drugs prescribed by generic name, the percentage of encounters where an antibiotic was prescribed, the percentage of encounters where an injection was the route of administration and the percentage of drugs prescribed from the Essential Drugs List (EDL) or another recognized formulary will be constructed.

Laboratory results like blood culture and white cell count will be classified as available (positive/Negative/unavailable) for blood culture or (normal (6-14), abnormal (<1 or >30), low (<6) or high(>25) for white blood cell count).

Blood culture results will be further categorised as positive with gram negative isolates sensitive or not sensitive to recommended 1^st^ line or 2^nd^ line antibiotics.

White blood cells will be categorised as normal if values are 6-14 or low if <6 and high if >14[13]

Clinical deterioration after 48 hours of admission based on new presence of any of the WHO danger signs listed above as a binary variable.

**Statistical methods**

**Participants**

A flow chart will detail the numbers in all sites (stratified by enrolment strata) of:

- Enrolled
- Withdrew or absconded treatment before discharge from the index admission.
- Died before discharge from the index admission.
- Discharged alive from the index admission.
- Still in hospital at 30 days
- Re-admitted to hospital within 30 days.

Baseline characteristics

Study participants characteristics at admission to hospital by enrolment strata will be described using N and proportions. For continuous variables, mean (sd) and median (IQR), depending on distribution (Table 1) will be reported for all sites combined.

Outcome data

The outcome will be reported as i) proportions of participants who received IV and oral antibiotics as 1^st^ line or 2^nd^ line IV antibiotics (as per WHO guidelines); and ii) receiving specific classes of antibiotics as per the WHO AWaRe antibiotic categories with binomial exact 95% confidence intervals.

Main results

Table of baseline characteristics (clinical and socio-demographic characteristics)

Table of the proportion of hospitalised children given any oral or IV antibiotics prior and during admission, the antimicrobials, classes and WHO AWaRe categories prescribed at admission or introduced after at least 48 hours.

Description of the number of children who restart antibiotics within 30 days of index admission including restarting in hospital and children who get readmitted.

Table of the distribution of antibiotics/combinations prescribed for children admitted stratified by nutritional groups, age and site.

Table of the number of hospital-days spent on each class of antibiotics (AWARE classifications), and the incidence of use of second and third-line antibiotics- – check for proportion of cases who had de-escalation.

Figure showing the number of days on prescribed 1st, 2nd and 3rd line IV antibiotics, number of deaths, withdrawals or discharged across days (example from work on clinical signs given below)

Table of exposures potentially associated with antibiotic classes and AWaRe categories

Multivariable regression analysis to determine factors associated with types of antibiotics prescribed (exposure variables are listed in section 3.7) as 1st line or 2nd line prescription, and the factors associated with receiving specific types of antibiotics (aminoglycosides, penicillin, beta lactams, macrolides, monobactams, fluoroquinolones, 2nd or 3rd generation cephalosporins).


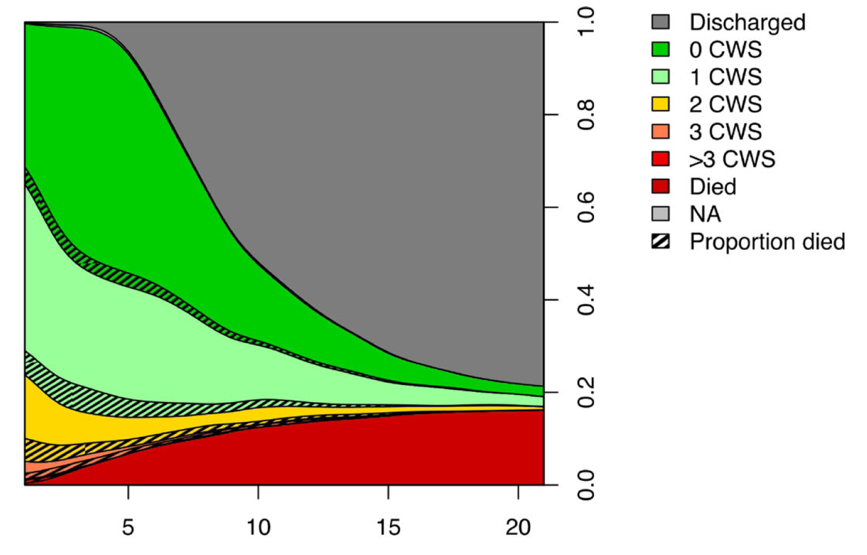
Example across days:

Summary of analyses

Descriptive statistics like prevalence, frequencies, means and standard deviations will be used to summarise patient characteristics and antimicrobial use patterns. Comparison of categorical variables will be done using Fishers exact and Pearson’s chi-square. Incidence rates for the change of antibiotics used to 2^nd^ line and 3^rd^ line will be as the sum of all new episodes of change to a higher-level antibiotic.

Regression analysis

Appropriate multilevel binomial regression models will be used to identify factors associated with the antibiotics prescribed during hospitalisation (as 1^st^ line or 2^nd^ line) and prescribed specific types of antibiotics. The multilevel binomial models will include recruiting sites as random intercept and inverse sampling weights. Appropriate feature selection methods will be used to select exposures to retain in the multivariable regression models. The variability of antibiotic prescribing, explained by the exposure variables will be measured using R-Squared.

References

1. Organization WH: **How to investigate drug use in health facilities: selected drug use indicators**. In*.*: World Health Organization; 1993.

2. Demoz GT, Kasahun GG, Hagazy K, Woldu G, Wahdey S, Tadesse DB, Niriayo YL: **Prescribing Pattern of Antibiotics Using WHO Prescribing Indicators Among Inpatients in Ethiopia: A Need for Antibiotic Stewardship Program**. *Infection and Drug Resistance* 2020, **13**:2783.

3. Shiva F, Ghanaie R, Shirvani F, Armin S, Tabatabaei SR, Fahimzad SA, Fallah F, PourMoshtagh H, Karimi A: **Pattern of antibiotic usage in children hospitalized for common infectious diseases**. *Archives of Pediatric Infectious Diseases* 2018, **6**(1).

4. Gerber JS, Kronman MP, Ross RK, Hersh AL, Newland JG, Metjian TA, Zaoutis TE: **Identifying targets for antimicrobial stewardship in children's hospitals**. *Infection Control & Hospital Epidemiology* 2013, **34**(12):1252-1258.

5. Organization WH: **Antimicrobial resistance: global report on surveillance**: World Health Organization; 2014.

6. Paterson DL: **Resistance in gram-negative bacteria: Enterobacteriaceae**. *American journal of infection control* 2006, **34**(5):S20-S28.

7. Moxon CA, Paulus S: **Beta-lactamases in Enterobacteriaceae infections in children**. *Journal of Infection* 2016, **72**:S41-S49.

8. Smith R, Coast J: **The true cost of antimicrobial resistance: Richard Smith and Joanna Coast argue that current estimates of the cost of antibiotic resistance are misleading and may result in inadequate investment in tackling the problem**. *Bmj* 2013, **346**.

9. Naylor NR, Atun R, Zhu N, Kulasabanathan K, Silva S, Chatterjee A, Knight GM, Robotham JV: **Estimating the burden of antimicrobial resistance: a systematic literature review**. *Antimicrobial Resistance & Infection Control* 2018, **7**(1):58.

10. Ventola CL: **The antibiotic resistance crisis: part 1: causes and threats**. *Pharmacy and therapeutics* 2015, **40**(4):277.

11. Illness CA, Network N: **Childhood Acute Illness and Nutrition (CHAIN) Network: a protocol for a multi-site prospective cohort study to identify modifiable risk factors for mortality among acutely ill children in Africa and Asia**. *BMJ open* 2019, **9**(5):e028454.

12. Maina M, Mwaniki P, Odira E, Kiko N, McKnight J, Schultsz C, English M, Tosas-Auguet O: **Antibiotic use in Kenyan public hospitals: prevalence, appropriateness and link to guideline availability**. *International Journal of Infectious Diseases* 2020, **99**:10-18.

13. Kironde F, Sekikubo M, Naiwumbwe H, Namusoke F, Buwembo W, Kiwuwa S, Oketch B, Noor R, Chilengi R, Mworozi E: **Hematology and blood serum chemistry reference intervals for children in Iganga district of Uganda**. *Health* 2013, **2013**.
